# Abcb4 acts as multixenobiotic transporter and active barrier against chemical uptake in zebrafish (*Danio rerio*) embryos

**DOI:** 10.1186/1741-7007-11-69

**Published:** 2013-06-17

**Authors:** Stephan Fischer, Nils Klüver, Kathleen Burkhardt-Medicke, Mirko Pietsch, Anne-Marie Schmidt, Peggy Wellner, Kristin Schirmer, Till Luckenbach

**Affiliations:** 1 Department of Environmental Toxicology, Eawag, Swiss Federal Institute of Aquatic Science and Technology, 8600 Dübendorf, Switzerland; 2 Department of Environmental Systems Sciences, ETH Zürich, Institute of Biogeochemistry and Pollutant Dynamics, 8092 Zürich, Switzerland; 3 Department of Bioanalytical Ecotoxicology, UFZ – Helmholtz Centre for Environmental Research, 04318 Leipzig, Germany; 4 Institute of Hydrobiology, Dresden University of Technology, 01062 Dresden, Germany; 5 Laboratory of Environmental Toxicology, EPF Lausanne, School of Architecture, Civil and Environmental Engineering, 1015 Lausanne, Switzerland

**Keywords:** Abcb4, Abcb5, Chemosensitization, Efflux transporters, Environment-tissue barrier, Multixenobiotic resistance, MXR, P-glycoprotein

## Abstract

**Background:**

In mammals, ABCB1 constitutes a cellular “first line of defense” against a wide array of chemicals and drugs conferring cellular multidrug or multixenobiotic resistance (MDR/MXR). We tested the hypothesis that an ABCB1 ortholog serves as protection for the sensitive developmental processes in zebrafish embryos against adverse compounds dissolved in the water.

**Results:**

Indication for ABCB1-type efflux counteracting the accumulation of chemicals in zebrafish embryos comes from experiments with fluorescent and toxic transporter substrates and inhibitors. With inhibitors present, levels of fluorescent dyes in embryo tissue and sensitivity of embryos to toxic substrates were generally elevated. We verified two predicted sequences from zebrafish, previously annotated as *abcb1*, by cloning; our synteny analyses, however, identified them as *abcb4* and *abcb5*, respectively. The *abcb1* gene is absent in the zebrafish genome and we explored whether instead Abcb4 and/or Abcb5 show toxicant defense properties. Quantitative real-time polymerase chain reaction (qPCR) analyses showed the presence of transcripts of both genes throughout the first 48 hours of zebrafish development. Similar to transporter inhibitors, morpholino knock-down of Abcb4 increased accumulation of fluorescent substrates in embryo tissue and sensitivity of embryos toward toxic compounds. In contrast, morpholino knock-down of Abcb5 did not exert this effect. ATPase assays with recombinant protein obtained with the baculovirus expression system confirmed that dye and toxic compounds act as substrates of zebrafish Abcb4 and inhibitors block its function. The compounds tested comprised model substrates of human ABCB1, namely the fluorescent dyes rhodamine B and calcein-am and the toxic compounds vinblastine, vincristine and doxorubicin; cyclosporin A, PSC833, MK571 and verapamil were applied as inhibitors. Additionally, tests were performed with ecotoxicologically relevant compounds: phenanthrene (a polycyclic aromatic hydrocarbon) and galaxolide and tonalide (two polycyclic musks).

**Conclusions:**

We show that zebrafish Abcb4 is a cellular toxicant transporter and provides protection of embryos against toxic chemicals dissolved in the water. Zebrafish Abcb4 thus is functionally similar to mammalian ABCB1, but differs from mammalian ABCB4, which is not involved in cellular resistance to chemicals but specifically transports phospholipids in the liver. Our data have important implications: Abcb4 could affect bioavailability - and thus toxicologic and pharmacologic potency - of chemicals to zebrafish embryos and inhibition of Abcb4 therefore causes chemosensitization, that is, enhanced sensitivity of embryos to toxicants. These aspects should be considered in (eco)toxicologic and pharmacologic chemical screens with the zebrafish embryo, a major vertebrate model.

## Background

ATP-binding cassette (ABC) transporters are one of the largest membrane protein super-families and are present in all biota from prokaryotes to mammals. They play a fundamental role in the transport of exogenous and endogenous compounds across inter- and intracellular boundaries. Malfunction of certain members of the ABC family causes various diseases, such as Tangier disease, cystic fibrosis and Dubin-Johnson syndrome [1]. Among transporters that lower the cellular load of various toxicants and drugs and thus maintain physiological integrity is ABCB1^a^ or P-glycoprotein, the best studied ABC transporter thus far. It confers so-called multidrug resistance (MDR) by diverting a range of drugs away from their site of pharmacologic action, a central concern in drug design for cancer therapy. On the other hand, ABCB1-like activity that was found in a wide range of organisms provides protection from toxicants, granting so-called multixenobiotic resistance (MXR) [2]. Indeed, knock-out of *Abcb1* in mice led to an increased permeability of the blood–brain barrier for neurotoxic compounds, allowing for enhanced brain penetration and lethal effects [3]. Moreover, occurrence of aquatic species in polluted environments is linked to high expression and increased efflux activity of Abcb1 orthologs [4], which are constituents of the transporter-mediated “environment-tissue barrier” [5]. In fact, mammalian ABCB1 is regarded as the only ABC transporter that has no physiological function other than protection of cells against a wide range of chemicals [6]. In contrast, two other ABC transporters that are structurally highly similar to ABCB1, ABCB4 and ABCB5, appear not to be involved in cellular protection against toxicants. ABCB4 has a specific physiological function in the liver and transports only particular compounds [7,8]. Over-expression of ABCB5 in certain cancer cells is associated with multidrug resistance [9], but it may not provide toxicant protection to cells in its normal function.

*Ex utero* embryo development is common among aquatic organisms and requires cellular adaptations affording explicit robustness and protection against adverse environmental impacts. These “orphan” embryos are literally flooded with a multitude of natural and man-made chemicals and efflux transporter proteins appear to form a primary defense mechanism that keeps these compounds out [10]. For instance, embryos of the echiuroid worm, *Urechis caupo*, which develop in mudflats containing metabolic products of bacteria and plants, showed high levels of ABCB1-like efflux activity [11].

The zebrafish (*Danio rerio*) embryo has emerged as a valuable vertebrate model in diverse fields, such as genetics and human disease, pharmacology and (eco)toxicology [12]. While it represents the most diverse group of vertebrates - fishes - it also offers the exquisiteness of fast development, access to genetic manipulation, comparability of organogenesis to higher vertebrates and transparency, along with suitability for high-throughput screening. For example, 309 chemicals were evaluated for potential human health and ecological effects as part of the U.S. Environmental Protection Agency’s (EPA’s) ToxCast™ phase-I program ([13]; [14]) and thousands of chemicals were screened to identify novel neuroactive drugs in a high-throughput behavioral assay with zebrafish embryos [15]. Moreover, developments are underway to establish the zebrafish embryo for rapid evaluation of the toxicity and bioaccumulation potential of the thousands of chemicals undergoing environmental risk assessment instead of relying on juvenile and adult fish [16]. The role of ABC transporters in the zebrafish developmental processes and in dealing with chemical exposure is, however, as yet unknown, and uptake and distribution of chemicals are generally assumed to be driven by passive diffusion.

We here demonstrate that the uptake of chemicals by zebrafish embryos can in fact be substantially influenced by ABC transporter activity. Throughout the first 48 hours of development, zebrafish embryos exhibit activity of an ABCB1-like efflux. Surprisingly, however, an *abcb1* ortholog appears to be absent in zebrafish. We found gene expression of *abcb4* and *abcb5* in zebrafish embryos that both are structurally similar to mammalian *Abcb1* and we show that Abcb4, but not Abcb5, protein possesses functional properties of mammalian ABCB1, constituting an active barrier against chemical uptake and conferring resistance of embryos to ABCB1 substrates.

## Results and discussion

### P-glycoproteins in zebrafish are *abcb4* and *abcb5* orthologs

Three structurally similar proteins, ABCB1, ABCB4 and ABCB5, are considered as “P-glycoproteins”. The zebrafish possesses only two P-glycoprotein genes that, being annotated as *abcb1* orthologs, were previously designated *abcb1a* and *abcb1b*; *abcb4* and *abcb5* were considered absent [17]. We confirmed the predicted cDNA sequences of both genes by cloning. Nucleotide sequence identity of the cDNAs is 59% and the deduced amino acid sequences show 50 to 64% identities with human ABCB1, ABCB4 and ABCB5 (Additional file 1: Table S1).

**Figure 1:**
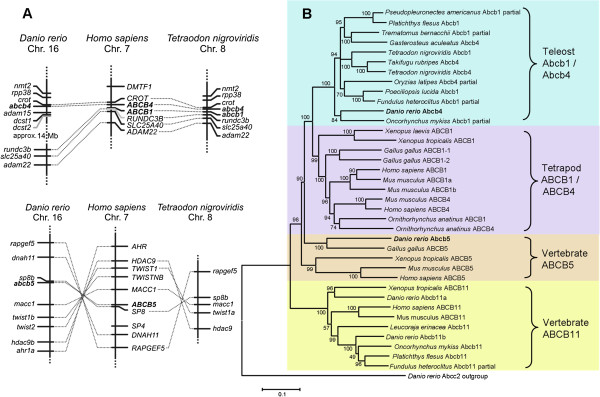
**Synteny and phylogenetic analysis of zebrafish *abcb4*/Abcb4 and *abcb5*/Abcb5. A)** Conserved synteny of zebrafish (*Danio rerio*), green spotted puffer (*Tetraodon nigroviridis*) and human (*Homo sapiens*) *abcb1/ABCB1*, *abcb4/ABCB4* and *abcb5/ABCB5* regions. The human *ABCB1*/*ABCB4* region is syntenic to zebrafish and green spotted puffer. The human *ABCB5* region is syntenic to zebrafish and green spotted puffer; however, green spotted puffer lacks a *abcb5* ortholog. **B)** The phylogenetic tree based on the multiple alignments (Clustal X2) of Abcb1, Abcb4, Abcb5 and Abcb11 amino acid sequences from *Danio rerio* and other vertebrates. The tree was generated using the neighbor-joining method and the percentage concordance based on 1,000 bootstrap iterations is shown at the nodes. Protein accession nos. (Ensembl or GenBank) are listed in Additional file 1: Table S2.

According to current annotations on the ensembl database [18] former *abcb1a* is designated *abcb5* (ENSDARG00000021787) and former *abcb1b* is *abcb4* (ENSDARG00000010936); an *Abcb1* ortholog is absent. Our synteny analyses of the respective chromosomal localizations of the genes in zebrafish are in line with the ensembl annotations and support that the zebrafish P-glycoproteins are not *abcb1* orthologs. Human *ABCB1* and *ABCB4* are consecutively located, whereas in zebrafish there is only one P-glycoprotein in a corresponding chromosome region. This gene is in close proximity to *crot*, resembling human *ABCB4* and *CROT* (Figure 1A). Furthermore, genes characteristic for the close vicinity of human and other vertebrate *Abcb1* genes, comprising *rundc3b, slc25a40 and adam22*, are located approximately 14 megabases (Mb) away from this locus in zebrafish. In concordance with zebrafish, other fish species (*Latimeria chalumnae, Gasterosteus aculeatus, Oryzias latipes, Gadus morhua*) also possess only *abcb4,* but not *abcb1* (Additional file 1: Figure S1), whereas *Takifugu rubripes* and *Tetraodon nigroviridis* have both *abcb1* and *abcb4* (Figure 1A, Additional file 1: Figure S1)*.* Analysis of the *abcb1a/abcb5* chromosomal localization indicates that zebrafish *abcb1a* is an *abcb5* ortholog. This chromosomal region of *abcb5* shares synteny with human *ABCB5* (Figure 1A), indicating that this locus has a common ancestral origin. Zebrafish Abcb1a/Abcb5 protein also associates with Abcb5 orthologs from other organisms in a phylogenetic tree (Figure 1B).

In this tree, proteins from different fish species designated as “Abcb4”, “Abcb1”, “Mdr1” or “P-glycoprotein” group to a cluster of fish P-glycoproteins that show close relationship with tetrapod ABCB1 and ABCB4 (Figure 1B). This suggests that tetrapod and teleost ABCB1/Abcb1 and ABCB4/Abcb4 have a common ancestor. Mammalian ABCB1 and ABCB4 sequences are highly similar but the functions of the proteins largely differ. Whereas substrate recognition of ABCB1 is rather unspecific, enabling it to act as a versatile efflux pump of toxicants, ABCB4 is a highly specialized transporter that trans-locates certain phospholipids and does not efflux toxicants from cells [7]. Differences in function despite the high sequence similarity of ABCB1 and ABCB4 raise the question if functional properties of the closely related fish P-glycoproteins are ABCB1- or ABCB4-like and if they are toxicant transporters. Further, the ability of human ABCB5 to confer multidrug resistance of cancer cells [9] may suggest that the zebrafish Abcb5 ortholog may act as a cellular efflux pump of toxicants.

The absence of an *abcb1* ortholog contradicted detection of an ABCB1-like efflux activity and multixenobiotic resistance of zebrafish embryos (see below). We, hence, investigated whether instead the other P-glycoproteins, Abcb4 and Abcb5, act as toxicant transporters.

### *Abcb4* and *abcb5* transcripts are constitutively expressed during the first 48 hours of zebrafish embryo development

We examined constitutive expression levels of *abcb4* and *abcb5* transcripts in zebrafish embryos from 1 to 48 hpf (hours post fertilization) for determining if these genes are transcribed and thus are candidates to be involved in cellular toxicant defense in these stages. Transcripts of both transporters were present at all sampled time points of development (1, 6, 12, 24, 48 hpf) (Table 1). *Abcb4* and *abcb5* transcript levels varied one to two orders of magnitude among developmental stages, but expression was generally low in comparison to, for example, *β-actin* with transcript levels that were three to four orders of magnitude higher. From the different embryonic stages considered transporter transcript levels of both transporters were highest at 1 hpf; *abcb4* was lowest at 12 hpf, increasing from there on; *abcb5* levels varied from stage to stage (Table 1). Transcripts in 1 and 6 hpf embryos are maternally transferred mRNA as *de novo* transcription is initiated between mid-blastula (1 k-cell stage: 3 hpf) and gastrula stages (75% epiboly; 7 hpf) [19].

**Table 1:** **Relative *abcb4 *and *abcb5 *mRNA abundances in zebrafish embryos at different developmental stages**

**hpf**	**1**	**6**	**12**	**24**	**48**
***abcb4***	**57.09 ± 13.03**	**2.36 ± 0.91**	**0.91 ± 0.26**	**2.03 ± 0.31**	**11.62 ± 0.99**
***abcb5***	**11.81 ± 1.87**	**2.05 ± 0.74**	**7.81 ± 0.74**	**2.36 ± 1.19**	**0.614 ± 0.09**
*β-actin*	20,575 ± 572	15,613 ± 6,938	24,639 ± 9,007	17,740 ± 3,948	22,004 ± 6,891

The mRNA abundances were determined in embryos at 1, 6, 12, 24 and 48 hours post fertilization (hpf). The expression levels that were normalized to *18S* rRNA are shown as means ± SD of four independent RNA isolations. Expression values were multiplied by 10^6^ for clarity of presentation. *Beta-actin* transcript levels are exemplarily shown for a highly expressed gene for comparison.

**Figure 2:**
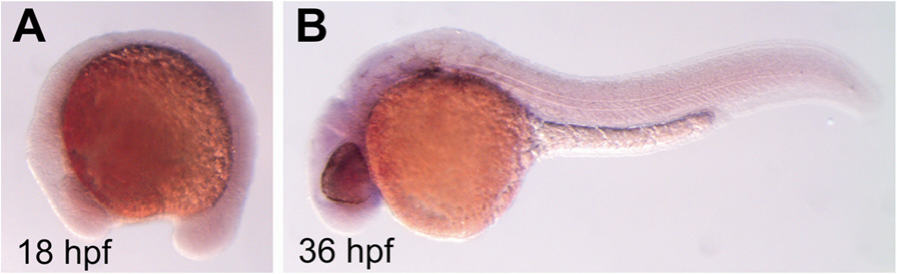
**Images of 18 hpf (A) and 36 hpf (B) zebrafish embryos showing *abcb4 *expression patterns.***Abcb4* transcripts where visualized with whole mount *in situ* hybridization. The faint but global staining patterns indicate weak, ubiquitous constitutive expression of *abcb4* in the embryos at both stages.

Whole-mount *in situ* hybridization (WISH) revealed ubiquitous, weak constitutive *abcb4* expression in the developmental period of zebrafish embryos examined here, which is indicated by faint purple staining of the entire embryos at the examined stages (Figure 2). Specificity of the probe we used was confirmed with 120 hpf embryos where a strong WISH signal occurred in the gut (Additional file 1: Figure S3). When assuming that transcript levels are indicative of protein expression and activity, the ubiquitous expression of *abcb4* transcripts found in 18 and 36 hpf embryos suggests a general continuous Abcb4 activity in all embryonic cells in this developmental phase.

Expression of *abcb5* transcripts has been found in epidermal cells of zebrafish embryos [20], which could point to a similar function of the protein as in mammals where it regulates membrane potential and cell fusion of skin progenitor cells [21].

### Abcb4 but not Abcb5 antagonizes accumulation of fluorescent transporter substrates in zebrafish embryos

Having shown that *abcb4* and *abcb5* transcripts are present in zebrafish embryos, we examined the function of the corresponding proteins as efflux pumps. Rhodamine B and calcein-am served as proxies for efflux transporter activity. Efflux transporter activity is indicated when the accumulation of fluorescent dye substrates is increased in cells due to disrupted transporter activity and, hence, disrupted active efflux of dye by transporter inhibiting chemicals [22] or by knock-down of the transporter protein.

#### Effects of transporter inhibitors on accumulation of rhodamine B and calcein-am/calcein in tissues of zebrafish embryos

**Figure 3:**
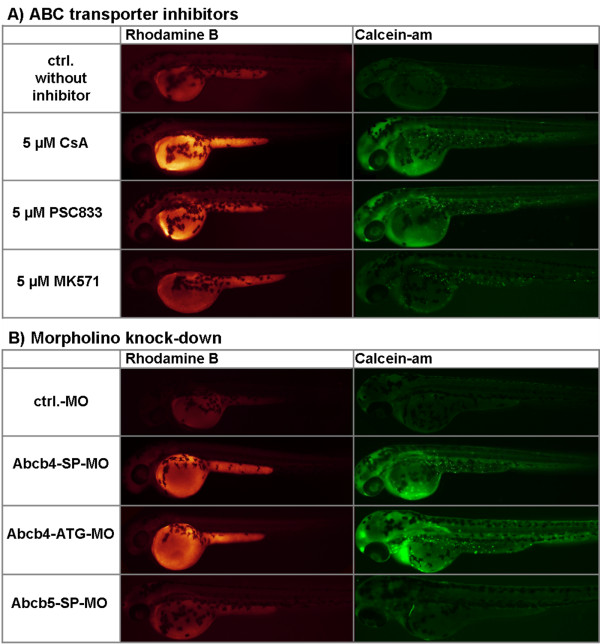
**Fluorescence micrographs of 48 hpf zebrafish embryos that had been incubated with 0.5 μM rhodamine B or 1.0 μM calcein-am either in the presence of different ABC transporter inhibitors (A) or upon knock-down of Abcb4 or Abcb5 (B).** ABC transporter inhibitor compounds were cyclosporin A (CsA), PSC833 and MK571. Morpholino oligonucelotides were used for knock-down of Abcb4 (Abcb4-SP-MO/Abcb4-ATG-MO) or Abcb5 (Abcb5-SP-MO). Exposure water of control embryos contained dye only (ctrl, ctrl-MO). The morpholino control (ctrl-MO) served to exclude unspecific morpholino effects on dye accumulation in the embryos. Brighter appearing embryos indicate accumulation of higher amounts of dye in the embryo tissue.

We measured changes in uptake of these dyes by embryos in the presence of two pharmacologic inhibitors of mammalian P-glycoproteins, namely cyclosporin A [23] and PSC833 [24], and of MK571, an inhibitor of mammalian ABCC transporters [25]. Fluorescence micrographs of embryos show that rhodamine B accumulated mainly in the yolk sac, whereas calcein fluorescence appeared in the head, trunk and cells scattered in the embryo body surface (Figure 3). Calcein-am is non-fluorescent, but once inside cells, it is hydrolyzed by cytosolic esterases and forms green, fluorescent calcein. Thus, calcein fluorescence in addition to efflux transporter activity also depends on the rate of calcein formation by esterases. Rhodamine B, on the other hand, is already fluorescent without modification in the cell and these differences may explain the differing spatial accumulation patterns of the dyes in embryo tissues. Fluorescence intensities of both dyes were increased when cyclosporin A, PSC833 or MK571 (Figure 3A) was also present, indicating elevated dye accumulation in the embryos. Thus, without an inhibitor, the accumulation of dyes in the embryos is kept low, evidencing that both rhodamine B and calcein-am are kept out of embryo tissues by an active efflux mechanism.

**Figure 4:**
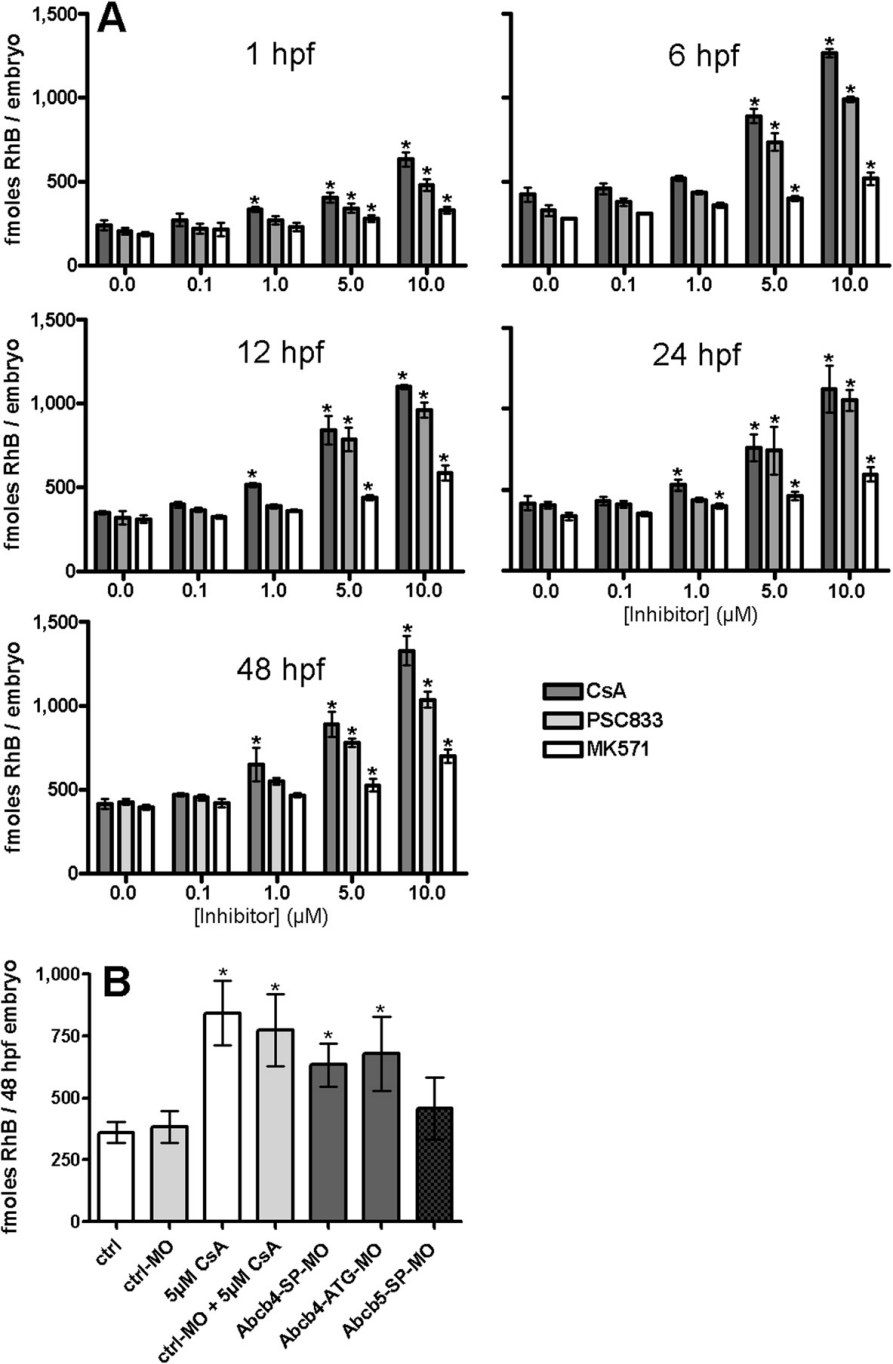
**Quantification of rhodamine B (RhB) dye accumulation in zebrafish embryos. (A)** Amounts of RhB accumulated in 1 hpf, 6 hpf, 12 hpf, 24 hpf and 48 hpf zebrafish embryos that, simultaneously to RhB, were exposed to various concentrations of transporter inhibitors cyclosporin A (CsA), PSC833 or MK571. **(B)** Amounts of RhB accumulated in 48 hpf zebrafish embryos upon morpholino knock-down of Abcb4 (Abcb4-SP-MO/Abcb4-ATG-MO) or Abcb5 (Abcb5-SP-MO). Controls contained dye only (ctrl, ctrl-MO), the morpholino control (ctrl-MO) served to exclude unspecific morpholino effects on dye accumulation in the embryos. Experiments with CsA (5 μM CsA, ctrl-MO + 5 μM CsA) were set up as comparisons for the effects of this pharmacologic transporter inhibitor on RhB accumulation in the embryos. Values are means +/− standard deviations from three independent experiments. Statistically significant differences from controls were determined with one-way analysis of variance (ANOVA), followed by Dunnett’s test and are indicated by asterisks (*P* <0.05).

For quantifying amounts of accumulated dye in embryos of different developmental stages, we measured rhodamine B levels in embryo tissue extracts following one hour of dye exposure. Rhodamine B amounts per embryo in controls, indicating the basal accumulation of the dye in the embryos with efflux transporters functioning, were approximately 200 fmol at 1 hpf, increasing to approximately 350 fmol at 6 hpf, 12 hpf and at 24 hpf and approximately 400 fmol at 48 hpf (Figure 4A, Additional file 1: Table S3). Enhanced accumulation of dye with the inhibitors cyclosporin A, PSC833 and MK571 was seen in embryos of all tested developmental stages, which demonstrates efflux transporter activities in all those stages. Rhodamine B tissue levels were elevated with increasing inhibitor concentrations and were up to 1.8- to 3.25-fold higher compared to controls (Figures 4A, Additional file 1: Table S3). For example, at 10 μM cyclosporin A or 10 μM PSC833, amounts of rhodamine B per embryo at 48 hpf were approximately 1,300 fmol and approximately 1,000 fmol, respectively. MK571 also increased dye accumulation in the embryos, but it was less potent than cyclosporin A and PSC833. Thus, at 10 μM MK571, rhodamine B amounted to approximately 700 fmol per 48 hpf embryo.

#### Effects of morpholino knock-down of Abcb4 and/or Abcb5 on accumulation of rhodamine B and calcein-am/calcein in tissues of zebrafish embryos

We examined whether Abcb4 and/or Abcb5 mediate cellular efflux activity in the embryos with morpholino knock-down. We used splice-blocking morpholinos for knock-down of Abcb4 (Abcb4-SP-MO, 0.5 mM) and Abcb5 (Abcb5-SP-MO, 0.5 mM) proteins, respectively, and assayed rhodamine B and calcein-am uptake by those embryos at 48 hpf. Control morpholino-treated embryos (Crtl-MO, 1 mM) served as the control for unspecific effects of morpholino injection on dye uptake. Abcb4 and Abcb5 knock-down did not cause phenotypic or necrotic effects, indicating that both Abcb4 and Abcb5 are not essential for development or basic physiological homeostasis in embryos up to 48 hpf. This is reminiscent of Abcb1 knock-out mice that also develop normally [3]. In the dye assays, rhodamine B and calcein fluorescence were brighter upon Abcb4 knock-down compared to the control, whereas Abcb5 knock-down had no visible effect on dye uptake (Figure 3B). Upon Abcb4 knock-down, approximately 650 fmol rhodamine B accumulated per 48 hpf embryo compared to approximately 450 fmol and approximately 400 fmol in Abcb5 knock-down and control morpholino treated embryos, respectively (Figure 4B). Abcb4 knock-down with a translation-blocking morpholino (Abcb4-ATG-MO, 0.0625 mM) resulted in accumulation of approximately 650 fmol rhodamine B per embryo, which is similar to the effect of the Abcb4 splice-blocking morpholino (Figures 3B and 4B). These results show that changes in rhodamine B or calcein accumulation were specifically related to Abcb4 knock-down. Thus, Abcb4, but not Abcb5, appears to act as an efflux pump of rhodamine B and calcein-am, respectively, keeping tissue concentrations of these compounds in zebrafish embryos low.

**Figure 5:**
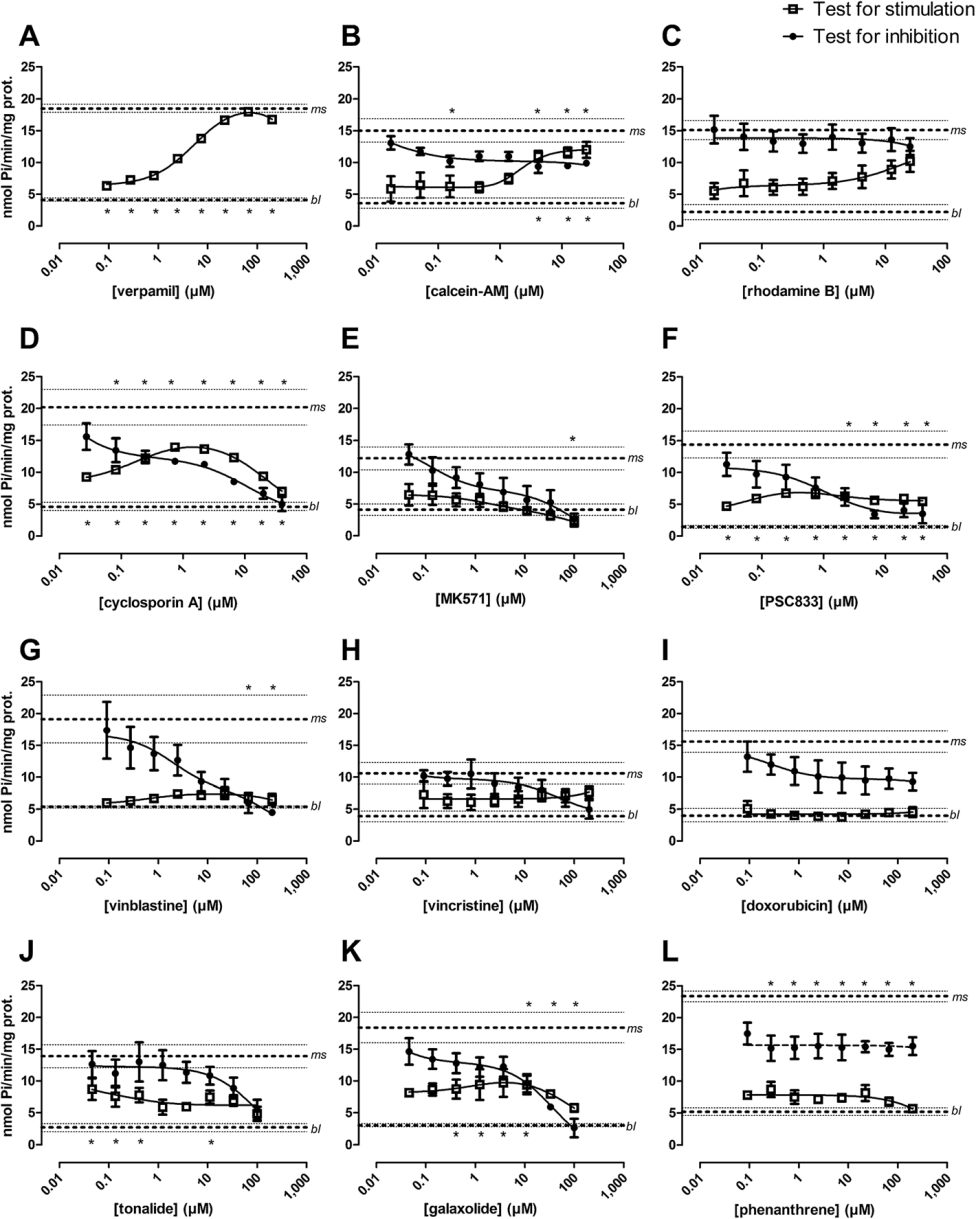
**Effects of chemicals on ATPase activities of recombinant zebrafish Abcb4.** Compounds were tested for stimulation (open squares) of Abcb4 ATPase and inhibition of the verapamil-stimulated Abcb4 ATPase activities (closed circles). Tested compounds were verapamil **(A)**, calcein-am **(B)**, rhodamine B **(C)**, cyclosporin A **(D)**, MK571 **(E)**, PSC833 **(F)**, vinblastine **(G)**, vincristine **(H)**, doxorubicin **(I)**, tonalide **(J)**, galaxolide **(K)** and phenanthrene **(L)**. Values are means +/− SEM from three to four independent experiments. Means and SEM ranges of the baseline (*bl*) and with 40 μM verapamil maximally stimulated ATPase activities (*ms*) are indicated as horizontal dotted lines. Regression curves for ATPase stimulation were fitted using a modified Michaelis-Menten equation {VS = (K_1_K_2_V_0_ + K_2_V_1_S + V_2_S^2^)/(K_1_K_2_ + K_2_S + S^2^) [28,29]}; regression curves for inhibition of the stimulated ATPase activities were fitted to a modified version of this equation describing the descending part of the curve {VS = V_1_ + ((V_2_X)/(K_2_ + X))}. Statistically significant effects on ATPase stimulation or inhibition of stimulated ATPase activities were identified by comparison of data with baseline and maximally stimulated ATPase activities, respectively, using one-way analysis of variance (ANOVA), followed by Dunnett’s test and are indicated by asterisks (*: *P* <0.05) below the baseline line (different from baseline activity - significant stimulation) or above the line of maximal stimulation (different from the maximally stimulated activity - significant inhibition).

From these data, efflux of rhodamine B can clearly be associated with Abcb4 activity; however, the effect of Abcb4 knock-down on rhodamine B accumulation in the embryos was not as pronounced as with high cyclosporin A and PSC833 concentrations (Figure 4A). The reason for this discrepancy of Abcb4 knock-down and inhibitor effects could be that morpholino knock-down does not result in complete suppression of Abcb4 protein function. Thus, morpholino treatment that leads to transient knock-down of correct pre-mRNA splicing or mRNA translation does not necessarily result in complete elimination of functional protein; thus, to a certain extent, functional Abcb4 may still have been present in Abcb4 knock-down embryos. Rhodamine B efflux could, apart from Abcb4, be mediated also by other transporters that remain functional in Abcb4 knock-down embryos, but are blocked by chemical inhibitors. There is, for example, indications that cyclosporin A and PSC833, apart from Abcb, also block activity of Abcc transporters of fish [26]. In addition to Abcb4, Abcc transporters that have earlier been shown to be expressed in zebrafish embryos [27] may play a role in rhodamine B efflux. However, our data indicate that the role of Abcc transporters in efflux of rhodamine B is minor: MK571, an inhibitor of mammalian ABCCs, has a comparatively small effect on rhodamine B efflux (Figures 3A and 4A). In addition, the MK571 effect on rhodamine B accumulation may in fact be Abcb4 inhibition as is indicated by our tests with MK571 and recombinant Abcb4 (Figure 5E, see below). These results thus support that rhodamine B efflux in zebrafish embryos is majorly mediated by Abcb4 and that Abcb4 knock-down does not completely disrupt Abcb4 function.

#### Effects of other chemicals on rhodamine B efflux in zebrafish embryos

**Figure 6:**
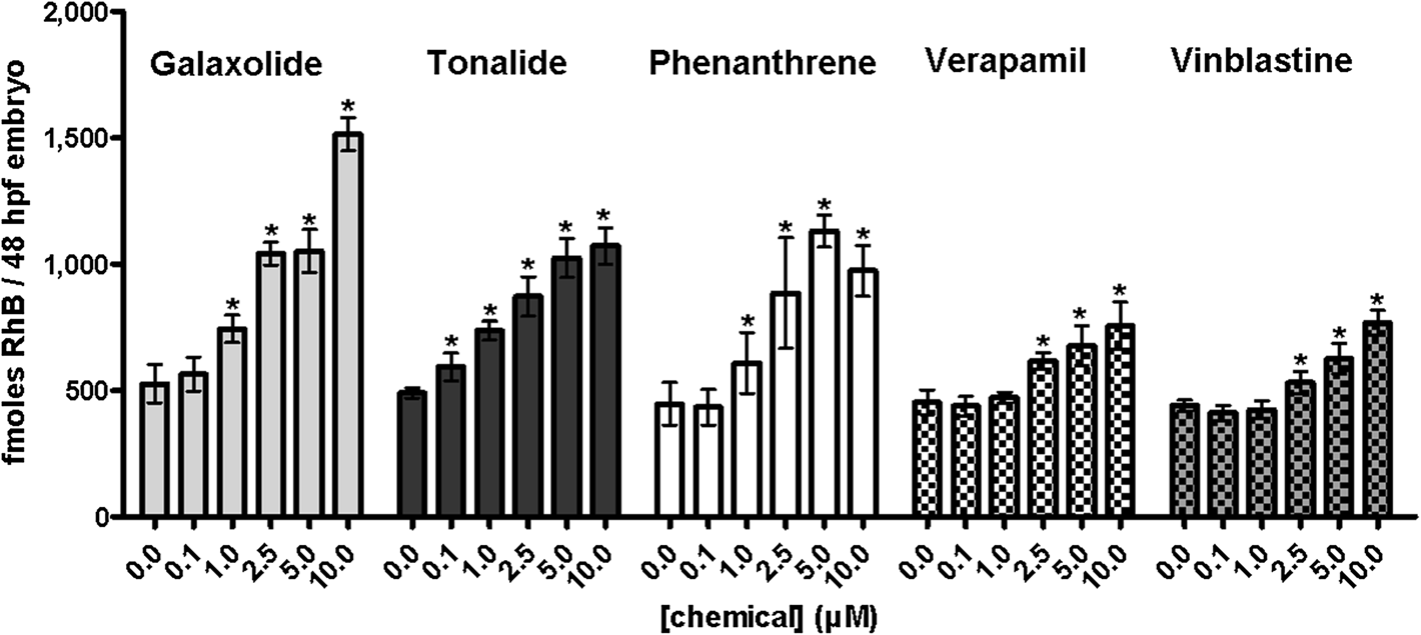
**Amounts of rhodamine B (RhB) accumulated in 48 hpf zebrafish embryos when also exposed to different concentrations of various chemicals.** These compounds comprised the synthetic musk fragrances galaxolide and tonalide, the polycyclic aromatic hydrocarbon phenanthrene, the ABCB1 inhibitor verapamil and the ABCB1 substrate vinblastine. Galaxolide, tonalide and phenanthrene are ecotoxicologically relevant chemicals. Embryos were exposed to RhB and simultaneously to either of these compounds. Values are means +/− standard deviations from three independent experiments. Statistically significant differences from controls were determined with one-way analysis of variance (ANOVA), followed by Dunnett’s test and are indicated by asterisks (*P* <0.05).

Galaxolide, tonalide, phenanthrene, verapamil and vinblastine also caused increased accumulation of rhodamine B in 48 hpf zebrafish embryos in a concentration-dependent manner (Figure 6, Additional file 1: Table S3). The data confirm interaction of those compounds with the rhodamine B efflux mechanism in zebrafish embryos that, as we show here, is Abcb4. Levels of rhodamine B in embryo tissue were significantly increased in zebrafish embryo tissue at concentrations ≥0.1 μM tonalide, ≥1 μM phenanthrene and galaxolide, and ≥2.5 μM verapamil and vinblastine (Figure 6, Additional file 1: Table S3). Galaxolide, tonalide and phenanthrene, which were included in the tests as ecotoxicologically relevant chemicals, were as effective as the inhibitors cyclosporin A and PSC833 causing accumulation of approximately 1,100 to 1,500 fmol rhodamine B per embryo, equivalent to 2.4- to 2.9-fold increases compared to the controls. Galaxolide and tonalide, polycyclic musk compounds, have earlier been found to act as efflux transporter inhibiting chemosensitizers in marine bivalves [30]; the data in this study indicate that the musks also affect the efflux of rhodamine B in zebrafish embryos, which appears to be conferred by Abcb4 (Figure 5J-K, see below). The effect of verapamil, a potent inhibitor of mammalian ABCB1, was not as high as was found with the other ABCB1 inhibitors, cyclosporin A and PSC833. The amount of rhodamine B was maximally 860 fmol per embryo, corresponding to a 1.9-fold increase of rhodamine B accumulated in embryos with verapamil present (Figure 6, Additional file 1: Table S3). Interestingly, however, our assays with recombinant protein indicated strong interaction of verapamil with Abcb4 from zebrafish, suggesting that verapamil acts as a substrate of this transporter (Figure 5A, see below). Being a substrate of the efflux transporter, verapamil may cause competitive inhibition of the transporter function and, therefore, its inhibitory potency depends on the degree of interference with another substrate (in our case rhodamine B) when both bind to the substrate binding site of the protein. Thus, interference can be low if two compounds bind to different sites of the substrate binding site [31]. The discrepancy of comparatively weak inhibition of rhodamine B efflux and strong interaction with the transporter protein ATPase by verapamil could thus be explained with little interference of the compounds when binding to the Abcb4 binding site. All of the tested compounds interacted with recombinant Abcb4 in the ATPase assay (Figure 5; see below) suggesting that their effect on rhodamine B accumulation in the embryos is associated with inhibition of the Abcb4-mediated efflux activity of rhodamine B.

### Disruption of Abcb4 activity leads to increased sensitivity of embryos to toxic transporter substrates

#### Effects of ABC transporter inhibitors on the mortality of embryos due to toxic compounds

We chose vinblastine, vincristine and doxorubicin, cytotoxic substrates of human ABCB1 [32-34], and phenanthrene as an ecotoxicologically relevant model compound, for determining to what extent chemical resistance of zebrafish embryos is associated with ABC transporter efflux activity. In initial experiments that served to determine concentrations of the compounds that were toxic to zebrafish embryos we focused on the micromolar concentration range in which interaction of chemicals with transporters is generally observed. When embryos were exposed to the compounds from 1 to 48 hpf, we found lethal effects of vinblastine at concentrations >1 μM and 100% mortality at concentrations ≥5 μM; of vincristine at concentrations ≥10 μM; and of phenanthrene at concentrations >1 μM and 100% mortality at concentrations ≥20 μM. Within the concentration range tested (up to 40 μM), vincristine did not cause 100% mortality. Toxicity of doxorubicin for zebrafish embryos appears to be low; we found no toxic effects for the compound at concentrations in the micromolar range and, indeed, lethal effects were reported for substantially higher concentrations [35].

In further experiments that served to explore the role of transporter activity for the sensitivity of zebrafish embryos to toxic compounds, the test compounds were applied in a concentration series with two concentrations of vincristine (20, 40 μM) that were found to be toxic and for vinblastine and phenanthrene within the range causing up to 100% mortality in zebrafish embryos; doxorubicin was not further considered in these experiments. Toxicities of vinblastine, vincristine and phenanthrene were compared when applied alone and in combination with the non-toxic concentration of 5 μM of the transporter inhibitor cyclosporin A and, in the case of vinblastine, as well with 5 μM PSC833.

**Figure 7:**
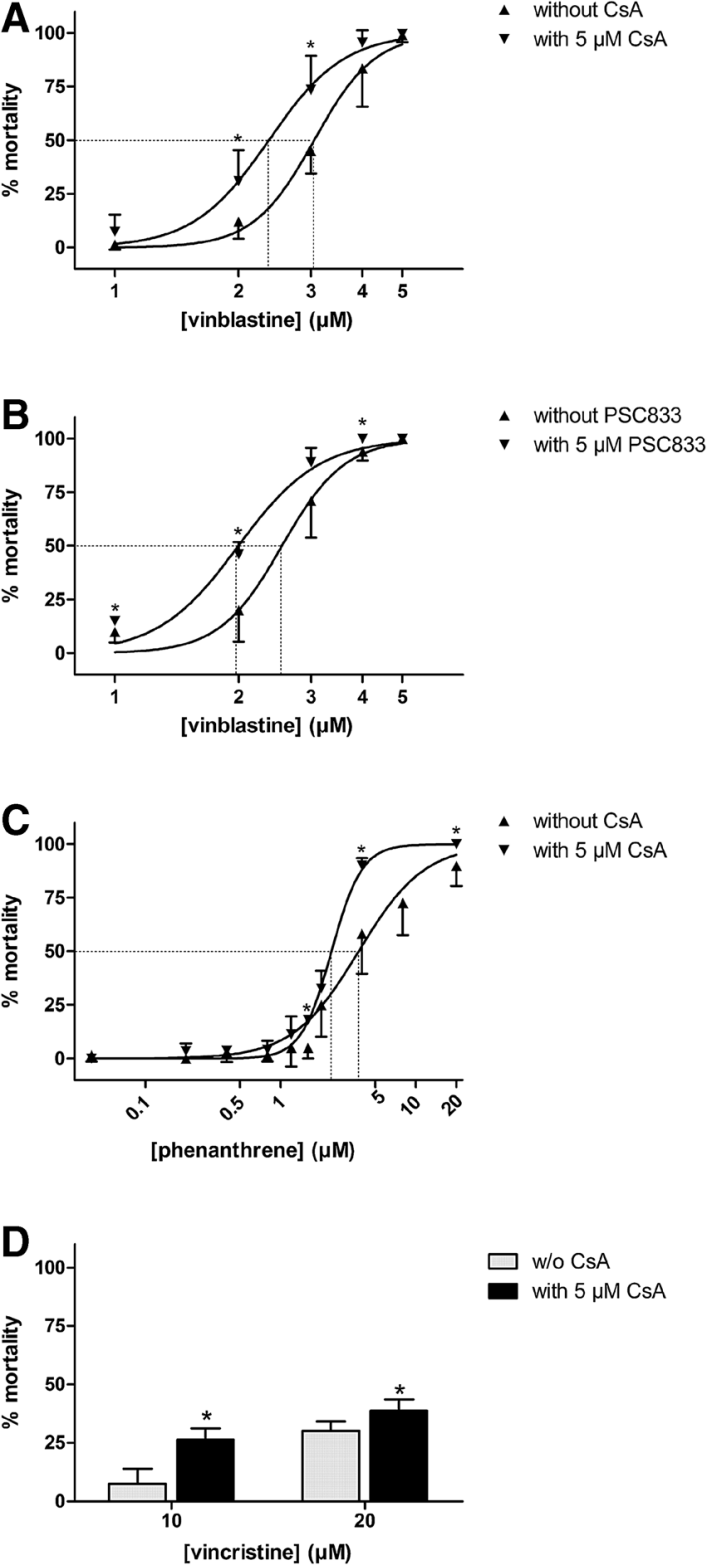
**Percent mortalities of zebrafish embryos after 1 to 48 hpf exposures to toxic compounds in the presence or absence of ABC transporter inhibitors.** The graphs show mortalities caused by different concentrations of vinblastine without and with 5 μM CsA **(A)** or 5 μM PSC833 **(B)** and of phenanthrene **(C)** and of vincristine **(D)** without and with 5 μM CsA. Depicted are mean values and standard deviations of independent replicates. For vinblastine and phenanthrene concentration-response regressions were determined using the logistic HILL model with bottom restrained to 0 and top restrained to 100. The dotted lines indicate the concentration causing mortality in 50% of the embryos (LC50). Refer to Additional file 1: Table S3 for details on the number of independent replicates, LC50 values with confidence intervals and further curve parameters. Statistically significant differences in mortalities between treatments without and with inhibitors at respective vinblastine, vincristine or phenanthrene concentrations were determined with the paired (vinblastine, vincristine) or the regular t-test (phenanthrene) and are indicated by asterisks (*P* <0.05).

**Figure 8:**
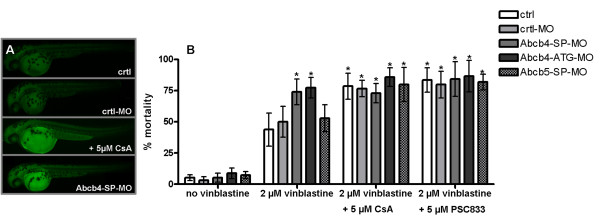
**Effects of morpholino knock-down of Abcb4 or Abcb5 and of transporter inhibitors on accumulation of bodipy-vinblastine and vinblastine-caused mortalities in zebrafish embryos. A)** Fluorescence micrographs depicting 48 hpf zebrafish embryos that had been incubated with bodipy-vinblastine either in combination with the inhibitor compound cyclosporin A (CsA) or upon knock-down of Abcb4 (Abcb4-SP-MO). The control was treated with dye only (ctrl), the morpholino control (ctrl-MO) served to exclude unspecific morpholino effects on dye accumulation in the embryos. **B)** Mortalities of zebrafish embryos upon treatment with morpholino nucleotides for knock-down of Abcb4 (Abcb4-SP-MO/Abcb4-ATG-MO) or Abcb5 (Abcb5-SP-MO) that were not exposed to any chemical (no vinblastine) or exposed to 2 μM vinblastine alone or in combination with 5 μM cyclosporin A (CsA) or 5 μM PSC833. Values are means +/− standard deviations from three to five independent experiments. The control morpholino (ctrl-MO)/2 μM vinblastine treatment served as reference for determining whether changes in mortality caused by 2 μM vinblastine were statistically significant when Abcb4 or Abcb5 were knocked-down or Abcb4 or Abcb5 knock-down was combined with CsA or PSC833 treatments. Significant differences were identified using one-way analysis of variance (ANOVA), followed by Dunnett’s test and are indicated by asterisks (*P* <0.05).

In an experimental series with vinblastine and cyclosporin A, LC50 values (concentration that was lethal for 50% of embryos) for vinblastine after exposure from 1 to 48 hpf were 3.05 μM (95% CI (confidence interval): 2.94 to 3.17 μM) without and 2.37 μM (95% CI: 2.25 to 2.49 μM) with cyclosporin A (Figure 7A), which is a difference of 22.3% (Additional file 1: Table S4). A similar decrease in LC50 for vinblastine was seen with PSC833 (Figure 7B) indicating higher toxicity of vinblastine when the transporter inhibitors were present. For testing whether the increase in vinblastine toxicity was indeed due to higher accumulations of the compound in the embryos, we studied uptake of bodipy-labeled, fluorescent vinblastine by embryos. Fluorescence intensities were elevated in embryos treated with 1.0 μM bodipy-vinblastine combined with cyclosporin A compared to embryos exposed to bodipy-vinblastine only (Figure 8A). This indicates that accumulation of bodipy-vinblastine in the embryos depends on efflux transporter activity and confirms that higher vinblastine toxicity in the presence of inhibitors is due to reduced efflux and thus increased accumulation of the compound in the embryos.

As found for vinblastine, mortalities of embryos were significantly increased when vincristine and phenanthrene, respectively, were combined with 5 μM cyclosporin A, likewise indicating higher accumulation of those compounds in the embryos when cellular efflux activity is blocked (Figures 7C, D). Mortalities caused by 10 and 20 μM vincristine were increased by 3.5- and 1.3-fold when cyclosporine was also present (Figure 7D). Cyclosporin A had a significant effect on the toxicity of phenanthrene, evidenced by a 37% difference between the LC50 values for phenanthrene alone (3.8 μM) and with cyclosporine (2.4 μM) (Figure 7C, Additional file 1: Table S3).

#### Effects of morpholino knock-down of Abcb4 or Abcb5 on vinblastine-caused mortalities in embryos

We furthermore examined the effect of disruption of transporter activity on resistance of embryos to vinblastine using morpholino knock-down. After 48 hours, exposure to 2 μM vinblastine caused 74% ± 10.2 mortality in embryos upon knock-down of Abcb4, whereas Abcb5 knock-down and control morpholino embryos showed mortalities (53% ± 10.6 and 50% ± 12.3, respectively) similar to untreated controls (ctrl; 44% ± 13.2 ) (Figure 8B). As with the Abcb4 splice-blocking morpholino treated embryos, elevated sensitivity to vinblastine was also found for embryos treated with the Abcb4 translation-blocking morpholino (Figure 8B). The experiments with bodipy-vinblastine support the interpretation that stronger effects by vinblastine upon Abcb4 knock-down are associated with increased vinblastine accumulation in the embryos. Thus, as with cyclosporin A, the bodipy-vinblastine fluorescence in embryos was likewise increased when Abcb4 knock-down embryos (Figure 8A). When non-morpholino, control morpholino or Abcb5 knock-down embryos were co-treated with cyclosporin A or PSC833, mortalities by 2 μM vinblastine were raised to similar levels as those in embryos with Abcb4 knock-down without an inhibitor. The similar effects of Abcb4 knock-down and of transporter inhibitor compounds indicate that Abcb4 has a major role in mitigating vinblastine toxicity in zebrafish embryos and that the sensitivity-enhancing effect of the inhibitors, cyclosporin A and PSC833, is blocking activity of this transporter. The effect in treatments with Abcb4 knock-down and inhibitors in combination tended to be slightly stronger than in treatments with only Abcb4 knock-down or inhibitors (Figure 8B). This can be seen as an indication that neither Abcb4 knock-down nor treatment with the inhibitors at the concentration applied lead to complete suppression of Abcb4 function, which is in concordance with the findings in the rhodamine dye accumulation experiments (see above, Figure 4B).

### Abcb4 substrates and inhibitors modulate the ATPase activity of recombinant zebrafish Abcb4

Our results from the dye uptake and toxicological assays indicate that (1) zebrafish embryos employ a multidrug transporter like cellular efflux mechanism that blocks uptake of chemicals by the embryos from the water and thus mitigates their toxic effects; (2) this cellular efflux of compounds is largely based on the activity of Abcb4, but not of Abcb5; and (3) effects of transporter inhibitors are associated with inhibition of Abcb4 activity.

Further evidence for interaction of the tested compounds with Abcb4 as substrates and/or inhibitors comes from assays where effects of chemicals on the ATPase activity of recombinant Abcb4 protein were quantified. As a measure for interaction of chemicals with Abcb4, changes in ATPase activity of recombinant Abcb4 with the tested chemicals present were used (Figure 5). Stimulation of the basal transporter ATPase activity, indicated as the amount of inorganic phosphate (Pi) released during the hydrolysis of ATP, occurs with compounds acting as substrates; inhibition of the stimulated ATPase activity is found for transporter inhibitors [36]. For stimulation of Abcb4 in the inhibition experiment and as a positive control we used verapamil, which stimulated the Abcb4 ATPase activity by 4.4-fold (mean of positive controls from all experiments). The action of verapamil on zebrafish Abcb4 is comparable to its effect on the human ABCB1 ATPase activity, which is stimulated by 4.5- to 5.0-fold [36,37].

#### Stimulation of the basal Abcb4 ATPase activity

The compounds that were applied as fluorescent (rhodamine B, calcein-am) or toxic (vinblastine, vincristine, phenanthrene) transporter substrates in experiments with the zebrafish embryos generally stimulated the basic Abcb4 ATPase activity (Figure 5B, C, G, H, L) although the effect was statistically significant only for calcein-am (Figure 5B). However, the Pi levels were generally above baseline also with rhodamine B, vinblastine, vincristine and phenanthrene (Figure 5C, G, H, L), which can be seen as an indication that these compounds also stimulated the Abcb4 ATPase activity, albeit to a lesser extent. Doxorubicin stimulates the ABCB1 ATPase activity only at very high concentrations [37] and, accordingly, the compound had no effect on the zebrafish Abcb4 ATPase activity in the concentration range we tested (Figure 5I).

Apart from the classic ABCB1 inhibitor verapamil, the other tested inhibitors, cyclosporin A and PSC833, significantly and, in the case of MK571, weakly, stimulated the Abcb4 ATPase activity (Figure 5D-F). The ATPase stimulating effect of cyclosporin A is consistent with its property as substrate of the mammalian ABCB1 [38], even though, in contrast to our results with Abcb4, the compound does not stimulate the ABCB1 ATPase [37]. Interestingly, substantial Abcb4 ATPase stimulation was seen with tonalide and galaxolide, indicating their properties as Abcb4 substrates (Figure 5J, K). However, as the compounds are very lipophilic [39], they probably highly accumulate in the cellular membranes and it is, therefore, questionable if Abcb4 activity leads to efflux of the compounds from the cells.

Activation of Abcb4 ATPase appeared in the nano- to low micro-molar concentration range of compounds corresponding to the concentrations applied in the dye uptake and toxicological assays with zebrafish embryos. This implies that these concentrations were in a range where the compounds are actively transported by Abcb4.

In the case of cyclosporin A, the concentration-dependent Abcb4 ATPase activity showed an initial increase in activity, followed by an activity decrease at higher concentrations (Figure 5D). This biphasic response in ATPase activities to increasing concentrations of a compound is common for substrates of human ABCB1 and it is associated with the presence of two binding sites with different binding affinities in the protein, one for stimulation (with a high affinity that is activated at low concentrations) and one for inhibition (with a low affinity that is activated at higher concentrations) [28]. A decrease in ATPase stimulation at higher concentrations was observed as a trend also for MK571, vinblastine, galaxolide, tonalide and phenanthrene (Figure 5E, G, J, K, L).

#### Inhibition of the stimulated Abcb4 ATPase activity

Except for rhodamine B (Figure 5C), all of the tested compounds caused a decrease in the stimulated Abcb4 ATPase activity and the results are congruent with the sensitizing effects of cyclosporin A and PSC833 in the embryotoxicity tests (Figures 7 and 8) and with the effects of cyclosporine A, PSC833, MK571, vinblastine, tonalide, galaxolide and phenanthrene on rhodamine B accumulation in embryo tissue (Figures 3A, 4 and 6). For MK571, which is an inhibitor of mammalian ABCC1, the inhibiting effect indicates that it also acts on zebrafish Abcb4 and, as discussed above, its effects on rhodamine B accumulation in the zebrafish embryos may in fact be from inhibition of Abcb4 in the embryos.

## Conclusions

We here show that cellular efflux activity of an Abcb4 ortholog in zebrafish embryos in the first 48 hours of development antagonizes uptake of chemicals from the water. This is evidenced by the finding that the presence of ABC transporter inhibitors as well as morpholino knock-down of expression of functional Abcb4 leads to increased accumulation of toxic and fluorescent transporter substrates in the embryos. ATPase assays with recombinant zebrafish Abcb4 confirm the properties of our test compounds as Abcb4 substrates and/or inhibitors. Since morpholino knock-down of Abcb4 and exposure of embryos to chemical transporter inhibitors had similar effects on accumulation of substrates in the embryos it can be concluded that zebrafish Abcb4 is a major component of the MXR system of zebrafish embryos.

This multixenobiotic resistance mediating function of the protein contrasts to mammalian ABCB4, which is a specialized translocator of phosphatidylcholine (PC) into bile that transports cytotoxic drugs only at low rates and does not confer multixenobiotic resistance [8]. Contrary to this, Abcb4 in fish may not act as a PC translocator as is indicated by the lack of PC in the bile of the Asian carp [40], which, like zebrafish, is a cyprinid. The ability to transport a wide range of toxicants is probably a primary, ancient property of P-glycoproteins that is conserved for Abcb4 in fish. This could explain why fish tolerate the absence of Abcb1 since function as a toxicant pump is executed by the Abcb4 ortholog. In adult zebrafish, Abcb1-like efflux activity has been found to be a component of the blood-brain barrier [41]. As Abcb1 is absent in zebrafish, this efflux activity may well be associated with the function of the Abcb4 ortholog.

Abcb5, the other P-glycoprotein in zebrafish, appears not to mediate xenobiotic resistance.

The Abcb4-mediated efflux activity of toxicants in zebrafish embryos, a major pharmacologic and toxicologic model system, has important implications: 1) Abcb4 activity can substantially determine bioavailability - and thus pharmacologic and toxicologic potency - of a diverse array of chemicals to zebrafish embryos; 2) chemicals that modulate Abcb4 activity, such as the chemical transporter inhibitors we used, will enhance bioavailability of chemicals to zebrafish embryos that are usually effluxed by Abcb4. This chemosensitization by efflux transporter inhibition can be caused by a variety of chemicals, including seemingly innocuous ones [42]. The polycyclic musk compounds galaxolide and tonalide are an example for such compounds as they do not exert obvious toxic effects on zebrafish embryo development [43], but they clearly act as potent Abcb4 inhibitors. Our data underscore that efflux transporters are underappreciated but are important determinants of bioavailability of chemicals to cells and organisms. On the other hand, chemicals can reverse transporter function and thus increase bioavailability of other compounds. Depending on efflux transporter interference with different compounds, bioavailability of compounds in a mixture can deviate from that of the same components when they are present alone. This suggests that current regulatory practice of assessing risks based on individual compounds may underestimate toxicity. In this context, our baculovirus-based Abcb4 activity assay has proven reliable to determine substrate/inhibitor properties of test compounds and will be useful in chemical screens using zebrafish.

## Methods

### Chemicals

Cyclosporin A, doxorubicin hydrochloride, phenanthrene, rhodamine B, verapamil hydrochloride and vincristine sulfate were from Sigma-Aldrich (Schnelldorf, Germany). Calcein-acetoxymethylester (calcein-am), MK571 and vinblastine sulfate were from Biozol (Eiching, Germany) and bodipy-vinblastine was from Invitrogen (Karlsruhe, Germany). PSC833 was a kind gift from Novartis (Basel, Switzerland). Galaxolide (73% of the total are diasterimeric isomers of 1,3,4,6,7,8-hexahydro-4,6,6,7,8,8-hexamethyl-cyclopenta-γ-[2]-benzopyran) was a kind gift from International Flavors & Fragrances Inc. (IFF; Union Beach, NJ, USA) and Tonalide (7-acetyl-1,1,3,4,4,6-hexamethyl-1,2,3,4-tetrahydronaphthalene; purity: 98%) was obtained from Bush Boake Allen Inc. (Jacksonville, FL, USA). RhB was dissolved in MilliQ water; stock solutions of all other chemicals were prepared in dimethyl sulfoxide (DMSO, Sigma-Aldrich). Final DMSO solutions in exposure media did not exceed 0.2%.

### Culture of zebrafish, collection of eggs and culture of embryos

Adult zebrafish from the WIK wildtype strain were maintained and bred according to standard protocols [44]. Collection of eggs and culturing of the embryos were performed as described [45].

### RNA extraction and reverse transcription

Total RNA was extracted from 30 to 50 embryos from 1, 6, 12, 24 and 48 hpf zebrafish embryos using *TRIzol* Reagent (Invitrogen) according to the manufacturer’s instructions. Genomic DNA contaminations were removed with DNAse I (Roche, Grenzach, Germany) treatment. cDNA was synthesized from total RNA using the High Capacity cDNA Reverse Transcription Kit (Applied Biosystems, Darmstadt, Germany).

### Cloning of zebrafish *abcb4* and *abcb5* cDNAs and phylogenetic and synteny analysis

Zebrafish *abcb4* (predicted sequence accession no. ENSDARG00000010936) and *abcb5* (predicted ENSDARG00000021787) sequences were obtained using reverse transcription polymerase chain reaction (RT-PCR) with primer pairs designed either based on the predicted sequences or based on sequences obtained with rapid amplification of cDNA ends (RACE) (Clontech, Palo Alto, USA). For PCR, Advantage 2 (Clontech) and Phusion (Finnzymes, Thermofisher, Schwerte, Germany) polymerases were used. PCR products were gel purified, cloned and sequenced on an ABI 3100 sequencer (Applied Biosystems) using standard cycle sequencing protocols. Sequences were edited and assembled using Sequencher 4.9 (Genecodes, Ann Arbor, USA) and analyzed using the National Center for Biotechnology Information (NCBI) basic alignment search tool (BLAST) and the Expert Protein Analysis System (ExPASy, Swiss Institute of Bioinformatics, Lausanne, Switzerland) [46]. The zebrafish *abcb4* and *abcb5* sequences were submitted to GenBank (NCBI). Accession numbers are listed in Additional file 1: Table S2.

Identity rates of zebrafish transporter nucleotide/amino acid sequences with vertebrate orthologs were determined with ClustalX2 (Conway Institute UCD, Dublin, Ireland). Phylogentic trees were generated with MEGA5 (Center for Evolutionary Medicine and Informatics, Tempe, USA) using the neighbor-joining method with percentage concordance based on 1,000 bootstrap iterations. To establish syntenic relationships between vertebrate genomes within the chromosomal regions of interest, we made use of ortholog predictions in the Ensembl database [18].

### Quantification of mRNA expression levels in zebrafish embryos

mRNA expression levels of *abcb4* and *abcb5* in 1, 6, 12, 24 and 48 hpf zebrafish embryos were quantified with quantitative RT-PCR (qPCR) using the SYBR Green PCR Master Mix (Quantace, Berlin, Germany). with an iCycler Real-Time PCR Detection System (BioRad, Munich, Germany).

Primers for housekeeping and zebrafish ABC transporter genes (Additional file 1: Table S5) were designed against available mRNA sequences from Ensembl [18] and self-obtained sequences using Beacon Designer (Primer Biosoft, Palo Alto, USA). Samples were run in triplicate in optically clear 96-well plates (Biozym, Hessisch Oldendorf, Germany). PCR was performed with RNA extracts from three different zebrafish embryo batches. qPCR results were calculated relative to the housekeeping gene, *18S* (for selection of the housekeeping gene refer to Additional file 1: Figure S2), according to the normalization procedure of the *Q-Gene Core Module*[47-49], which takes varying PCR amplification efficiencies into account (Additional file 1: Table S6). All qPCR experiments were performed according to the MIQE (Minimum Information for Publication of Quantitative Real-Time PCR Experiments) guidelines [50]. A MIQE checklist is found in Additional file 1.

### Whole-mount *in situ* hybridization

For whole-mount *in situ* hybridization (WISH), *abcb4* cDNA fragments were amplified (for primers refer to Additional file 1: Table S7), cloned into pCRII (Invitrogen) and verified by sequencing. WISH with 18, 38 and 120 hpf (functional proof of the used probes, specific staining in the intestine, see Additional file 1: Figure S3) zebrafish embryos was performed as described previously [51]. WISH staining was analyzed with a stereomicroscope (MZ16F, Leica, Wetzlar, Germany).

### Procedure for measuring efflux transporter protein activity in zebrafish embryos with fluorescent dyes

Fluorescent dyes, rhodamine B [22,52], calcein-am [53,54] and bodipy-vinblastine [55], served as proxies for efflux transporter activity in the fish embryos. When this activity is absent due to pharmacologic transporter inhibition or the absence of functional protein due to morpholino knock-down, accumulation of dye in the embryo tissue increases, resulting in a stronger fluorescence signal. Solutions for exposures were prepared in zebrafish embryo culture water with 0.5 μM rhodamine B, 1 μM calcein-am and 1 μM bodipy-vinblastine and inhibitors cyclosporin A (CsA), PSC833 and MK571, respectively. Up to 10 embryos per mL were incubated in the test solutions for one hour at 26°C in the dark, rinsed three times with clean culture water to remove dye from the chorion and subsequently photographed with a fluorescence microscope DMI 4000B (Leica) and DFC 350 FX camera (Leica). For quantification of RhB dye uptake, 10 embryos per treatment were sonicated in 200 μL of a hypotonic lysis buffer (10 mM KCl, 1.5 mM MgCl_2_, 10 mM Tris HCl, pH 7.4), the sonicates were briefly centrifuged, 150 μL of the supernatant were transferred to a black 96-well microplate (Nunc, Sigma-Aldrich, Schnelldorf, Germany) and the rhodamine B fluorescence was measured at 595 nm (emission)/530 nm (excitation) in a GENios plus fluorescence plate reader (Tecan, Männedorf, Switzerland). This assay enabled parallel examination of multiple treatments. Triplicates of five treatments along with a solvent control were run per experiment. Each experiment was repeated with embryos from three different egg batches laid on different days. The amount of rhodamine B accumulated in zebrafish embryos was quantified with a rhodamine B standard curve (Additional file 1: Figure S4).

### Embryo toxicity experiments

For determining toxicities of vinblastine, vincristine and doxorubicin, 20 embryos were incubated in glass petri dishes with 10 mL test solutions and two to three replicates per treatment. Exposures to phenanthrene were set up in tightly closed glass vials containing 2 mL solution with four embryos per vial according to Schreiber *et al*. [56] to prevent volatilization of phenanthrene from the test solutions. Per tested treatment, five vials were set up in parallel. Exposures were started with 4- to 16-cell stage embryos to assure successful fertilization and terminated after 48 hours. Exposure experiments were repeated with at least three batches of embryos from different days. During exposure, embryos were regularly examined using a stereo microscope and dead embryos were removed and recorded. A final mortality count was performed at 48 hours and embryos were declared as dead if at least one of the following criteria applied: i) coagulation of eggs, ii) no heart beat, iii) no blood circulation, iv) no somites, v) tail not detached [45]. Initially, toxic concentration ranges of vinblastine, vincristine, doxorubicin and phenanthrene and the transporter inhibitors CsA and PSC833 were identified. Subsequently, effects of transporter inhibitors at non-toxic concentrations and, in the case of vinblastine, of morpholino knock-down of Abcb4 and Abcb5 on the sensitivity of the embryos to toxic test compounds were determined. Controls contained i) 0.2% DMSO used as solvent, ii) inhibitors only or iii) morpholino knock-down embryos only. Mean mortality percentages and standard deviations at 48 hpf were calculated from all experimental replicates and the paired *t*-test was applied to determine whether inhibitors or morpholino knock-down significantly modulated vinblastine sensitivity of embryos.

### Production of recombinant zebrafish Abcb4 protein with the baculovirus expression system and ATPase activity measurements

The zebrafish *abcb4* cDNA was sub-cloned into pFastBac1 (Invitrogen) and sequenced for confirmation. *Abcb4* baculovirus was generated using the Bac-to-Bac Baculovirus Expression System (Invitrogen). Sf9 cells cultured in Sf-900 II SFM (Invitrogen) were used for virus amplification and protein expression. Crude Sf9 membranes with Abcb4 protein were prepared 60 hours after infection according to [57] and then stored at −80°C until use. Total protein in the membrane preparations was quantified using the bicinchoninic acid assay (BCA) and bovine serum albumin (BSA) as standard and the presence of Abcb4 protein in the membranes was checked in 1 μg of total protein by Western blotting using the anti-MDR1 antibody C219 (Additional file 1: Figure S5) as described previously [58].

ATPase assays were performed as described in [37] with minor modifications. Instead of 37°C, incubations of the membranes with the test compounds were performed for 40 minutes at 27°C, which is within the physiological temperature range of zebrafish. A total of 20 μg of protein was used for each reaction. The ATPase stimulating effect of verapamil, a classical stimulating agent of the mammalian Abcb1 ATPase, was also detected for zebrafish Abcb4 (Figures 5A) and 40 μM verapamil were used as positive control in ATPase stimulation assays and as ATPase stimulating agent in the ATPase inhibition assays. DMSO was used as the solvent for all compounds. The final DMSO concentration in the reaction was 2%, which did not affect ATPase activities.

### Abcb4 and Abcb5 knock-down by morpholino (MO) microinjection

Embryos were injected (FemtoJet, Eppendorf, Hauppauge, USA) through the chorion into the yolk compartment at the two-cell stage. Injection needles were pulled from borosilicate glass capillary tubes with filament (Warner Instruments, Hamden, USA) using a micropipette puller (Narishige, Tokyo, Japan).

Morpholinos (Gene Tools, Philomath, USA) were dissolved in MilliQ water and injected at the following concentration ranges: 0.5 mM to 2 mM Abcb4-MO-SP (SP = splice-blocking) (5′-AAT CTA ACT GCA TGA CGT ACT CTG T-3′); 0.0625 mM to 1 mM Abcb4-ATG-MO (translation-blocking) (5′-GCA AAC ATG GGC AAG AAA TCC AAA C-3′), 0.5 mM to 2 mM Abcb5-MO-SP (5′- GCA ACA GGT ACA TTC ATG TCT TTC T-3′) and 1 mM to 2 mM control splice morpholino (ctrl-MO) (MO against human beta-globin) (5′- CCT CTT ACC TCA GTT ACA ATT TAT A-3′). Functionality of Abcb4-SP-MO and Abcb5-SP-MO morpholinos were proven by means of RT-PCR (Additional file 1: Figure S6) and Abcb4-ATG-MO morpholino with co-injected Abcb4-ATG-GFP mRNA (Additional file 1: Figure S6). Injected embryos were cultured for ≤48 h and transporter activity dye assays and toxicity experiments were done as described above.

## Endnotes

^a^Our designation of gene and protein names is based on the Zebrafish Nomenclature Guidelines [59]; fish: *shh*/Shh, human: *SHH*/SHH, mouse: *Shh*/SHH (gene/protein).

## Abbreviations

ABC: ATP-binding cassette; BCA: bicinchoninic acid assay; BLAST: basic alignment search tool; BSA: bovine serum albumin; CsA: cyclosporin A; DMSO: dimethyl sulfoxide; hpf: hours post fertilization; EPA: Environmental Protection Agency; ExPASy: Expert Protein Analysis System; LC50: concentration that was lethal for 50% of embryos; Mb: megabases; MDR: multidrug resistance; MIQE: Minimum Information for Publication of Quantitative Real-Time PCR Experiments; MXR: multixenobiotic resistance; NCBI: National Center for Biotechnology Information; PC: phosphatidylcholine; Pi: inorganic phosphate; qPCR: Quantitative real-time polymerase chain reaction; RACE: rapid amplification of cDNA ends; WISH: whole-mount *in situ* hybridization.

## Competing interests

None of the contributing authors has any competing interests.

## Authors’ contributions

SF designed the study, performed experiments, analyzed data and wrote the paper. NK and KBM performed experiments, analyzed data and wrote the paper. MP, AMS and PW performed experiments and analyzed data. KS provided guidance to SF, analyzed data and wrote the paper. TL designed the study, provided guidance to SF, MP, KBM, AMS and PW, performed experiments, analyzed data and wrote the paper. All authors read, contributed feedback to, and approved the final manuscript.

## Authors’ information

Kristin Schirmer and Till Luckenbach are senior authors.

## Supplementary Material

Additional file 1 **Additional information regarding the qPCR analysis procedure: MIQE (Minimum Information for Publication of Quantitative Real-Time PCR Experiments) checklist. Table S1** with percent similarity data from sequence comparisons. The table is supplementary to Figure 1B. **Table S2** with the accession nos. of sequences used for phylogenetic analyses. The table is supplementary to Figure 1B. **Table S3** with RhB amounts that had accumulated in 1, 6, 12, 24 and 48 hpf zebrafish embryos upon co-exposure to various compounds. The table is supplementary to Figures 4 and 6. **Table S4** with values from concentration-effect curves determined in zebrafish embryo toxicity experiments. The table is supplementary to Figure 7. **Table S5** with sequences of primers used for qPCR. The table is supplementary to Table 1. **Table S6** with efficiencies of zebrafish *abcb4*, *abcb5* and housekeeping primers used in qPCR reactions. The table is supplementary to Table 1. **Table S7** with primer pairs used for PCR of zebrafish *abcb4* fragments used for generating probes for whole-mount *in situ* hybridization (WISH). The table is supplementary to Figure 2. **Figure S1** with conserved synteny of *abcb1/ABCB1* and *abcb4/ABCB4* regions in various species. **Figure S2** with Ct values determined for housekeeping gene candidates in different embryo stages of zebrafish with qPCR. The figure is supplementary to Table 1. **Figure S3** with images of 120 hpf zebrafish embryos with *abcb4* mRNA transcripts visualized using WISH. The figure is supplementary to Figure 2. **Figure S4** with a standard curve used to determine the amount of RhB taken up by zebrafish embryos. The figure is supplementary to Figures 4 and 6. **Figure S5** with images of Western blots with recombinant zebrafish Abcb4 protein obtained with the baculovirus expression system. **Figure S6** with results of experiments proving the functionality of the used morpholinos. Click here for file
