# Supplementary material for: Abcb4 acts as multixenobiotic transporter and active barrier against chemical uptake in zebrafish (Danio rerio) embryos
Source: BMC Biol. 2013 Jun 17;11:69. doi: 10.1186/1741-7007-11-69 (PMC3765700; doi:10.1186/1741-7007-11-69)
Supplement: Additional file 1 — Additional information regarding the qPCR analysis procedure: MIQE (Minimum Information for Publication of Quantitative Real-Time PCR Experiments) checklist. Table S1 with percent similarity data from sequence comparisons. The table is supplementary to Figure 1B. Table S2 with the accession nos. of sequences used for phylogenetic analyses. The table is supplementary to Figure 1B. Table S3 with RhB amounts that had accumulated in 1, 6, 12, 24 and 48 hpf zebrafish embryos upon co-exposure to various compounds. The table is supplementary to Figures 4 and 6. Table S4 with values from concentration-effect curves determined in zebrafish embryo toxicity experiments. The table is supplementary to Figure 7. Table S5 with sequences of primers used for qPCR. The table is supplementary to Table 1. Table S6 with efficiencies of zebrafish abcb4, abcb5 and housekeeping primers used in qPCR reactions. The table is supplementary to Table 1. Table S7 with primer pairs used for PCR of zebrafish abcb4 fragments used for generating probes for whole-mount in situ hybridization (WISH). The table is supplementary to Figure 2. Figure S1 with conserved synteny of abcb1/ABCB1 and abcb4/ABCB4 regions in various species. Figure S2 with Ct values determined for housekeeping gene candidates in different embryo stages of zebrafish with qPCR. The figure is supplementary to Table 1. Figure S3 with images of 120 hpf zebrafish embryos with abcb4 mRNA transcripts visualized using WISH. The figure is supplementary to Figure 2. Figure S4 with a standard curve used to determine the amount of RhB taken up by zebrafish embryos. The figure is supplementary to Figures 4 and 6. Figure S5 with images of Western blots with recombinant zebrafish Abcb4 protein obtained with the baculovirus expression system. Figure S6 with results of experiments proving the functionality of the used morpholinos. [file 1741-7007-11-69-S1.docx]

ADDITIONAL FILE 1.

**“Abcb4 acts as multixenobiotic transporter and active barrier against chemical uptake in zebrafish (*Danio rerio*) embryos”**

Stephan Fischer, Nils Klüver, Kathleen Burkhardt-Medicke, Mirko Pietsch, Anne-Marie Schmidt, Peggy Wellner, Kristin Schirmer, Till Luckenbach

**Contents:**

- Additional information regarding the qPCR analysis procedure
- Supplementary tables
- Supplementary figures

**Additional information regarding the qPCR analysis procedure:** Information of the performed qPCR analysis of zebrafish *abcb4* and *abcb5* mRNA expression based on the MIQE (Minimum Information for Publication of Quantitative Real-Time PCR Experiments) guidelines. All essential information (E) must be submitted with the manuscript. Desirable information (D) should be submitted if available.

|  | **IMPORTANCE** | **CHECKLIST** |
| --- | --- | --- |
| **EXPERIMENTAL DESIGN** |  |  |
| Definition of experimental and control groups | E | **√** |
| Number within each group | E | **√** |
| Assay carried out by core lab or investigator's lab? | D | Investigator |
| Acknowledgement of authors' contributions | D | **√** |
| **SAMPLE** |  |  |
| Description | E | **√** |
| Volume/mass of sample processed | D | **√** |
| Microdissection or macrodissection | E | No dissection |
| Processing procedure | E | **√** |
| If frozen -how and how quickly? | E | Not frozen |
| If fixed - with what, how quickly? | E | No fixation |
| Sample storage conditions and duration (especially for FFPE samples) | E | **√** |

For extraction of total RNA embryos from respective stages were pooled. Culture of zebrafish and embryos, treatment of zebrafish embryos, the number of zebrafish embryos pooled for RNA extraction and further details on the experimental design are provided below and in the Material and Methods section.

**NUCLEIC**

| **NUCLEIC ACID EXTRACTION** |  |  |
| --- | --- | --- |
| Procedure and/or instrumentation | E | **√** |
| Name of kit and details of any modifications | E | **√** |
| Source of additional reagents used | D | **√** |
| Details of DNase or RNAse treatment | E | **√** |
| Contamination assessment (DNA or RNA) | E | **√** |
| Nucleic acid quantification | E | **√** |
| Instrument and method | E | **√** |
| Purity (A260/A280) | D | **√** |
| Yield | D | **√** |
| RNA integrity method/instrument | E | **√** |
| RIN/RQI or Cq of 3' and 5' transcripts | E | **√** |
| Electrophoresis traces | D | **√** |
| Inhibition testing (Cq dilutions, spike or other) | E | **√** |

Total RNA was extracted from 30 to 50 pooled zebrafish embryos at 1, 6, 12, 24 and 48 hpf using *TRIzol* Reagent (Invitrogen) according to the manufacturer's instructions. The pooled embryos were homogenized in 0.5 mL *TRIzol* using an ULTRA-TURRAX homogenizer (IKA-Werke). The RNA from embryos at the different developmental stages from three different egg batches laid at different days was analyzed with qPCR. For removing traces of genomic DNA 4 μg of RNA was treated with 4 units of RNAse-free DNase (Roche) in a 40 μl final volume according to the manufacturer's instructions. RNA qualities and quantities were determined using a NanoDrop spectrophotometer (PEQLAB Biotechnologie GMBH). The quality of RNA with a 260/280 ratio between of 1.9-2.1 and a 260/230 ratio of 1.8-2.2 was considered satisfactory for use in our study. In addition, integrity of RNA from each extract was confirmed by inspecting the bands after electrophoresis of 1 µl RNA on a non-denaturing agarose gel. The extracted RNA was stored in eppendorf tubes at -80°C until further use.

Possible contaminations of the RNA from all samples were assessed with “no reverse transcription” by qPCR. Furthermore, a melting curve analysis was performed as standard in order to detect DNA contamination of the RNA which would be visible as further unspecific peak.

| **REVERSE TRANSCRIPTION** |  |  |
| --- | --- | --- |
| Complete reaction conditions | E | **√** |
| Amount of RNA and reaction volume | E | **√** |
| Priming oligonucleotide (if using GSP) and concentration | E | **√** |
| Reverse transcriptase and concentration | E | **√** |
| Temperature and time | E | **√** |
| Manufacturer of reagents and catalogue numbers | D | **√** |
| Cqs with and without RT | D* | **√** |
| Storage conditions of cDNA | D | **√** |

Reverse transcription was performed with the High Capacity cDNA Reverse Transcription Kit (Applied Biosystems). cDNA was synthesized from DNase-treated total RNA (1 μg). The 20 μl reaction contained 10 μl of RNA, 2.0 μl 10 X RT-Buffer, 2.0 μl 10XRT Random Primers, 0.8 μl 25XdNTP Mix (100 mM), 1.0 μl RNAse inhibitor, 1.0 μl MultiScribe Reverse Transcriptase and 3.2 Nuclease-free water. The Reaction Mix was incubated at 25°C for 10 min, then at 37°C for 120 min and finally at 85 °C for 5 min. The cDNA was stored in eppendorf tubes at -20°C until further analysis.

For the performed qPCR studies, in “no reverse transcription control samples” (RNA not treated with reverse transcription enzyme) no amplification was detected.

| **qPCR TARGET INFORMATION** |  |  |
| --- | --- | --- |
| If multiplex, efficiency and LOD of each assay. | E | only Singleplex |
| Sequence accession number | E | **√** |
| Location of amplicon | D | **√** |
| Amplicon length | E | **√** |
| *In silico* specificity screen (BLAST, etc) | E | **√** |
| Pseudogenes, retropseudogenes or other homologs? | D |  |
| Sequence alignment | D | **√** |
| Secondary structure analysis of amplicon | D | **√** |
| Location of each primer by exon or intron (if applicable) | E | NA |
| What splice variants are targeted? | E | NA |

Sequence accession numbers and amplicon lengths are listed in Table S3. The primer sets (Table S5) spanning intron regions of each gene were analyzed with NCBI Blast to ensure specificity.

| **qPCR OLIGONUCLEOTIDES** |  |  |
| --- | --- | --- |
| Primer sequences | E | **√** |
| RT Primer DB Identification Number | D |  |
| Probe sequences | D** | **√** |
| Location and identity of any modifications | E | NA |
| Manufacturer of oligonucleotides | D | **√** |
| Purification method | D | **√** |

Primers purified by desalting (DLS) were purchased from Invitrogen.

| **qPCR PROTOCOL** |  |  |
| --- | --- | --- |
| Complete reaction conditions | E | **√** |
| Reaction volume and amount of cDNA/DNA | E | **√** |
| Primer, (probe), Mg++ and dNTP concentrations | E | **√** |
| Polymerase identity and concentration | E | **√** |
| Buffer/kit identity and manufacturer | E | **√** |
| Exact chemical constitution of the buffer | D |  |
| Additives (SYBR Green I, DMSO, etc.) | E | SYBR Green I |
| Manufacturer of plates/tubes and catalog number | D | **√** |
| Complete thermocycling parameters | E | **√** |
| Reaction setup (manual/robotic) | D | **√** |
| Manufacturer of qPCR instrument | E | **√** |

qPCR analyses were carried out in optical 96-well plates (Biozym) in a iCycler Real-Time PCR Detection System (BioRad). Cycling parameters were as follows: 95 °C (10 min), 40 cycles of 95 °C (15 s), 55 °C (20 s) and 72 °C (20 s). A melting curve analysis was performed (95 °C for 15 s, 60 °C for 1min, 0.3 °C increases for 15 s up to 95 °C) after each run.

Samples contained 1× SYBR Green PCR Master Mix (Quantace), 0.5 µL of each primer (300 μM) and 2µL of cDNA template for a final reaction volume of 12.5 µL. Reactions were set up manually in a sterile bench using designated equipment.

| **qPCR VALIDATION** |  |  |
| --- | --- | --- |
| Evidence of optimisation (from gradients) | D |  |
| Specificity (gel, sequence, melt, or digest) | E | **√** |
| For SYBR Green I, Cq of the NTC | E | **√** |
| Standard curves with slope and y-intercept | E | **√** |
| PCR efficiency calculated from slope | E | **√** |
| Confidence interval for PCR efficiency or standard error | D |  |
| r2 of standard curve | E | **√** |
| Linear dynamic range | E | **√** |
| Cq variation at lower limit | E | **√** |
| Confidence intervals throughout range | D |  |
| Evidence for limit of detection | E | **√** |
| If multiplex, efficiency and LOD of each assay. | E | only Singleplex |

The specificity of the amplification products has been confirmed by size estimations on a

agarose gel, sequencing of the products and by analyzing their melting curves. Serial 10-fold

dilution of cDNAs were used to calculate the standard curve and measure the amplification efficiency for each target and all tested housekeeping genes (see Table S6).

| **DATA ANALYSIS** |  |  |
| --- | --- | --- |
| qPCR analysis program (source, version) | E | **√** |
| Cq method determination | E | **√** |
| Outlier identification and disposition | E |  |
| Results of NTCs | E | **√** |
| Justification of number and choice of reference genes | E | Only one |
| Description of normalisation method | E | **√** |
| Number and concordance of biological replicates | D | **√** |
| Number and stage (RT or qPCR) of technical replicates | E | **√** |
| Repeatability (intra-assay variation) | E | **√** |
| Reproducibility (inter-assay variation, %CV) | D | **√** |
| Power analysis | D |  |
| Statistical methods for result significance | E | **√** |
| Software (source, version) | E | **√** |
| Cq or raw data submission using RDML | D |  |

- qPCR analysis program: iQ5 Optical System *Software* Version 2.0 (BioRad)
- Obtained data were analyzed using the comparative Ct (threshold cycle) method.
- Cq’s were determined by setting the threshold automatically
- No data were excluded from the calculations
- Results of NTCs: no amplification products present thus no Cqs
- Justification of number and choice of reference genes: see Figure S2
- Description of normalization method: endogenous reference gene: see Material and Methods section
- Number and concordance of biological replicates: Four independent biological replicates were analyzed.
- Number and stage of technical replicates: three technical replicate reactions for each biological replicate

**Supplementary tables**

**Table S1.** Percent similarity matrix with *abcb4*/Abcb4 and *abcb5*/Abcb5 from zebrafish compared with representative orthologs from other vertebrates. Multiple sequence alignments of the nucleotide/amino acid sequences were performed using Clustal X. Accession nos. (nucleotide/amino acid sequence from Ensembl or Genbank) of ABC transporter sequences are listed in Table S2.


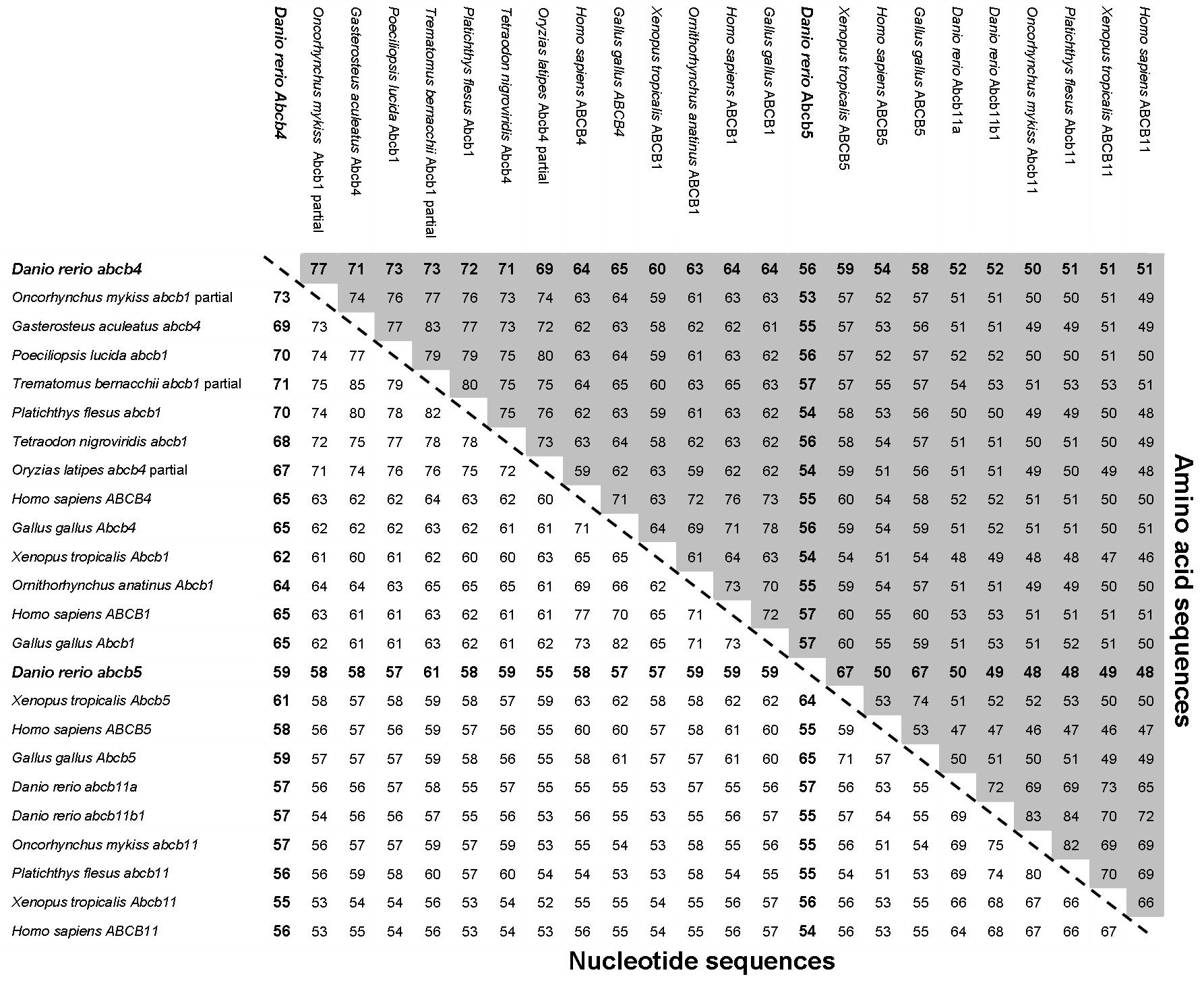


**Table S2.** ABC tranporter nucleotide and amino acid sequence accession nos. from Ensembl or Genbank for different vertebrate species. Sequences were used for phylogenetic and sequence identity analyses of zebrafish *abcb4*/Abcb4 and *abcb5*/Abcb5 transporters.

| **Species** | **ABC transporter**  **gene / protein name** | **Nucleotide sequence**  **accession no.** | **Amino acid sequence**  **accession no.** |
| --- | --- | --- | --- |
| ***Danio rerio***  (Zebrafish) | *abcb4* / Abcb4 | JQ014001 | AFR69055.1 |
|  | *abcb5* / Abcb5 | JQ014002 | AFR69056.1" |
|  | *abcb11a* / Abcb11a | XM_001337688.4 | XP_001337724.4 |
|  | *abcb11b1* / Abcb11b1 | XM_001923503.4 | XP_001923538.3 |
| ***Fundulus heteroclitus***  (Killifish) | *abcb1* / Abcb1 partial | AF099732.1 | AAD23956.1 |
|  | *abcb11* / Abcb11 partial | AF135793.1 | AAD29692.1 |
| ***Gallus gallus***  (Chicken*)* | *Abcb1* / ABCB1 | ENSGALG00000008912 | ENSGALG00000008912 |
|  | *Abcb4 /* ABCB4 | ENSGALT00000014467 | ENSGALP00000014451 |
|  | Abcb5 / ABCB5 | ENSGALT00000017732 | ENSGALP00000017711 |
| ***Gasterosteus aculeatus***  (Stickleback) | *abcb4* / Abcb4 | ENSGACT00000012310 | ENSGACP00000012286 |
| ***Homo sapiens***  (Human) | *ABCB1* / ABCB1 | NM_000927.4 | NP_000918.2 |
|  | *ABCB4* / ABCB4 | NM_000443.3 | NP_000434.1 |
|  | *ABCB5* / ABCB5 | NM_001163941.1 | AAP55848.1 |
|  | *ABCB11* / ABCB11 | NM_003742.2 | AAD28285.1 |
| ***Leucoraja erinacea***  (Skate) | abcb11 / Abcb11 | AF367243.1 | AAK52958.1 |
| ***Mus musculus***  (Mouse) | *Abcb1a* / ABCB1a | NM_011076.2 | NP_035206.2 |
|  | *Abcb1b* / ABCB1b | NM_011075.2 | NP_035205.1 |
|  | *Abcb4* / ABCB4 | NM_008830.2 | NP_032856.2 |
|  | *Abcb5* / ABCB5 | NM_029961.2 | NP_084237.1 |
|  | *Abcb11* / ABCB11 | NM_021022.3 | NP_066302.2 |
| ***Oncorhynchus mykiss***  (Rainbow trout) | *abcb1* / Abcb1 partial | AY863423.3 | AAW56424.3 |
|  | abcb11 / Abcb11 | NM_001124656.1 | NP_001118128.1 |
| ***Ornithorhynchus*** ***anatinus*** (Platypus) | *Abcb1* /ABCB1 | ENSOANT00000007005 | ENSOANP00000007003 |
|  | *Abcb4* / ABCB4 | ENSOANT00000007007 | ENSOANP00000007005 |
| ***Oryzias* *latipes*** (Medaka) | *abcb4* / Abcb4 partial | ENSORLT00000011623 | ENSORLG00000009269 |
| ***Platichthys flesus***  (European flounder) | *abcb1* / Abcb1 | AJ344049.1 | CAC86600.1 |
|  | *abcb11* / Abcb11 | AJ344042.1 | CAC86593.1 |
| ***Poeciliopsis lucida***  (Clearfin livebearer) | Abcb1 | DQ842514.2 | ADQ20481.1 |
| ***Pseudopleuronectes americanus***  (Winter flounder). | *abcb1* / Abcb1 partial | AY053461.1 | AAL15148.1 |
| ***Takifugu rubripes***  (Japanese pufferfish) | *abcb4* / Abcb4 | AF164138.1 | AAO20901.1 |
| ***Tetraodon nigroviridis***  (Green spotted pufferfish) | *abcb1* / Abcb1 | ENSTNIT00000000709 | ENSTNIP00000000891 |
|  | *abcb4* / Abcb4 | ENSTNIT00000000556 | ENSTNIP00000000474 |
| ***Trematomus bernacchii***  (Emerald rockcod) | *abcb1* / Abcb1 partial | FJ938210.1 | ACX30417.1 |
| ***Xenopus laevis***  (African clawed frog) | *Abcb1* / ABCB1 | NM_001087925 | NP_001081394.1 |
| ***Xenopus tropicalis***  (Western clawed frog) | *abcb1* / ABCB1 | ENSXETP00000005311 | ENSXETP00000005311 |
|  | *abcb5* / ABCB5 | ENSXETT00000016207 | ENSXETP00000016207 |
|  | *abcb11* / ABCB11 | XM_002936755.1 | XP_002936801.1 |

**Table S3.** Quantified amounts of RhB that had accumulated in zebrafish embryos after co-exposure to different concentrations of CsA, PSC833 and MK571 (1, 6, 12, 24, 48 hpf) and of galaxolide, tonalide, phenanthrene, verapamil and vinblastine (48 hpf).


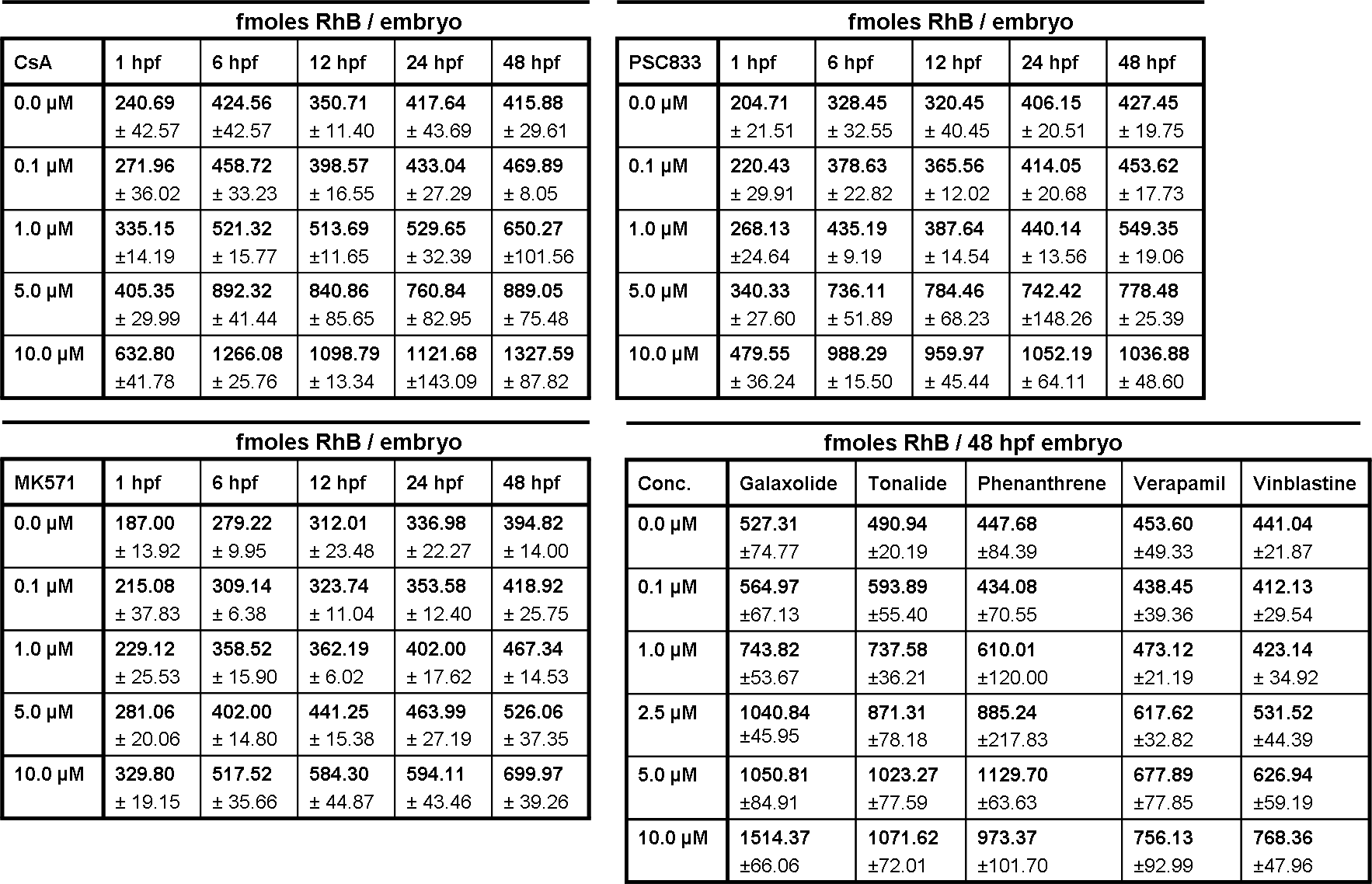


## Table S4. Calculated LC50 values with 95 % confidence intervals (CI) and the % differences of LC50s for zebrafish embryos after 1 to 48 hpf exposures to different concentrations of vinblastine with and without CsA or PSC833 and phenanthrene with and without CsA.

| **Treatment** | **LC50 (95 % CI) [µM]** | **curve fit** | **hill slope** | **N** |
| --- | --- | --- | --- | --- |
| vinblastine w/o CsA | 3.051 (2.94/3.17) | 0.94 | 5.858 | 10 |
| vinblastine with 5 µM CsA | 2.367 (2.25/2.49) | 0.92 | 4.721 |  |
| % difference of LC50s (with/without CsA) | 22.4 |  |  |  |
| vinblastine w/o PSC833 | 2.55 (2.39/2.72) | 0.9278 | 5.697 | 5 |
| vinblastine with 5 µM PSC833 | 2.00 (1.84/2.16) | 0.9198 | 4.419 |  |
| % difference of LC50s (with/without PSC833) | 21.7 |  |  |  |
| phenanthrene w/o CsA | 3.775 (3.34/4.27) | 0.8766 | 1.784 | 3 - 13 |
| phenanthrene with 5 µM CsA | 2.38 (2.15/2.63) | 0.8893 | 3.88 |  |
| % difference of LC50s (with/without CsA) | 36.9 |  |  |  |

**Table S5.** Primer pairs (F: forward, R: reverse) used for quantitative real time PCR of zebrafish *abcb4* and *abcb5* and housekeeping genes with amplicon length and NCBI accession nos. Housekeeping genes: *18s* - 18S ribosmal RNA; *bactin* - beta actin; *ef1a* - elongation factor1-alpha*,* *gapdh* ***-*** glycerinaldehyde-3- phosphat dehydrogenase*, b2m* ***-*** beta-2 microglobulin. 18S ribosomal RNA showed the most stable expression over the different zebrafish developmental stages (see Figure S1), therefore it was used as housekeeping gene for our quantitative real time PCR analysis.

| **Gene name** | **Primer Sequence**  **(5´-3´)** | **Amplicon**  **Length** | **NCBI**  **accession no.** |
| --- | --- | --- | --- |
| ***abcb4*** | F:TACTGATGATGCTTGGCTTAATC  R:TCTCTGGAAAGGTGAAGTTAGG | *159* | JQ014001 |
| ***abcb5*** | F) CGCTGGTCATTCTGGCTGTC  R) CTCCTCTGCTACCGCTCCAG | *125* | JQ014002 |
| ***18S*** | F: TCGCTAGTTGGCATCGTTTATG  R: CGGAGGTTCGAAGACGATCA | *162* | BX296557.35 |
| ***bactin*** | F: CGAGCAGGAGATGGGAAC  R: CGTGGATACCGCAAGATT | 158 | AF057040 |
| ***ef1a*** | F: TCAAGAAGATCGGCTACAAC  R: GGCAGAATGGCATCAAGG | *160* | NM_131263.1 |
| ***gapdh*** | F: AGGCAGAAGGCGGCAAAC  R: AAGACACCAGTAGACTCCACAAC | *124* | BC083506 |
| ***b2m*** | F: GCCTTCACCCCAGAGAAAGG  R: GCGGTTGGGATTTACATGTTG | l01 | BC062841 |

**Table S6.** Amplification efficiency for each target and all tested housekeeping genes

| **Gene** | **Efficiency** | ***SE(E)*** | ***Slope*** | **R^2^** | **NTC** |
| --- | --- | --- | --- | --- | --- |
| ***abcb4*** | 92.53 | *0.011* | -3.59 | *0.996* | *N/A* |
| ***abcb5*** | 104.14 | *0.009* | -3.19 | *0.998* | *N/A* |
| ***18S*** | 100.67 | *0.007* | -3.3 | *0.998* | *N/A* |
| ***bactin*** | 88.23 | 0.014 | -3.76 | 0.993 | *N/A* |
| ***ef1a*** | *94.12* | *0.010* | -3.53 | *0.995* | *N/A* |
| ***gapdh*** | 96.87 | *0.014* | *-3.43* | *0.994* | *N/A* |
| ***b2m*** | 105.31 | 0.017 | -3.15 | 0.995 | *N/A* |

**Table S7.** Primer pairs (F: forward, R: reverse) with amplicon lengths used for PCR of fragments of zebrafish *abcb4* (NCBI acc. no. JQ014001) for use as templates for whole-mount *in situ* hybridization (WISH) probes (Figure2).

|  | **Primer sequence (5´-3´)** | **Amplicon length (bp)** |
| --- | --- | --- |
| ***probe 1*** | F2: TGGGCAAGAAATCCAAACTC  R1: TGTCATCACCTTTCCGATGA | 725 |
| ***probe 2*** | F3: CCTCACAGATGAGCCACTGA  R3: TGTGTGCTAGGAAAACAGTGC | 564 |
| ***probe 3*** | F4: GCAGAGAAGTGGACCAGGAG  R4: CCCCATTACCTGTGGTATTTGA | 513 |

**Supplementary figures**


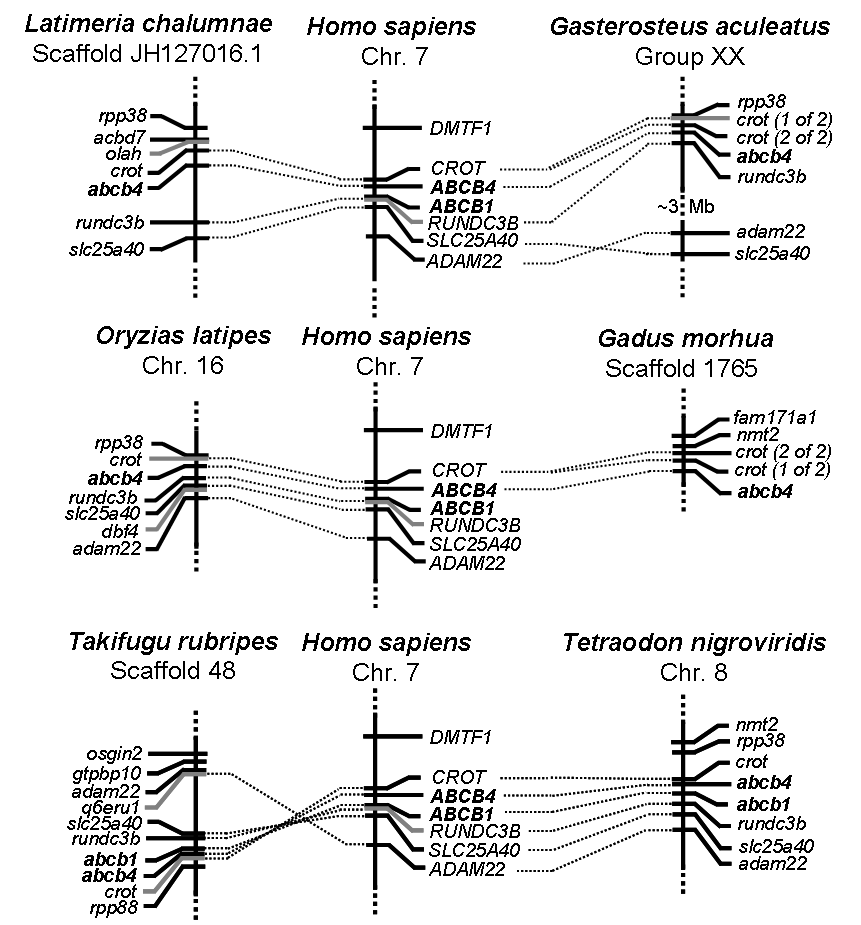


**Figure S1.** Conserved synteny of coelacanth *(Latimeria chalumnae),* stickleback *(Gasterosteus aculeatus),* medaka (*Oryzias latipes),* cod *(Gadus morhua)*, japanese pufferfish *(Takifugu* *rubripes),* green spotted pufferfish *(Tetraodon nigroviridis)* and human (*Homo sapiens)* *abcb1/ABCB1* and *abcb4/ABCB4* regions. Dotted lines illustrate that the human *ABCB1*/*ABCB4* region is syntenic to coelacanth, stickleback, medaka, cod, japanese pufferfish and green spotted pufferfish.

**Figure S2.** Levels of mRNA abundance of housekeeping gene candidates in different zebrafish embryo stages, quantified by qPCR. Ct values represent mean +/- SD from three independent RNA isolations of each stage. With the exception of 18S (differences in Ct among stages < ± 1), basal expression levels of genes varied among the different stages (differences of Ct > ± 2 for *bactin,* *gapdh,* *ef1a* and *b2m*). Based on these results 18s was chosen as housekeeping gene for the study.


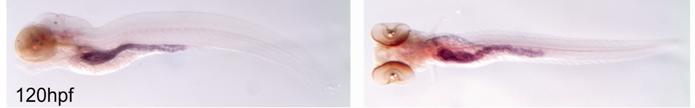


**Figure S3.** Images of 120 hpf zebrafish embryos in which *abcb4* mRNA transcripts where visualized with whole-mount *insitu* hybridization (WISH). The intestinal bulb and intestine are strongly stained at this stage showing high expression of *abcb4*. The images are provided here to confirm specificity of the *abcb4* WISH probes we used.


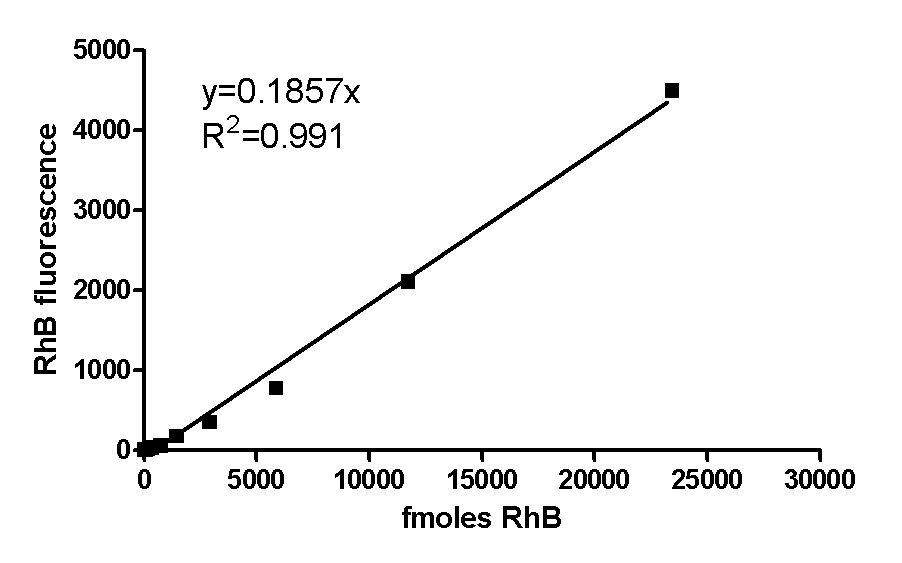


**Figure S4.** Rhodamine B (RhB) standard curve used for the calculation of the amount of RhB accumulated in zebrafish embryo tissue (Figure 4). Solutions of different concentrations of RhB were set up in the buffer used for RhB extractions from embryos. RhB fluorescence of extracts from a pool of ten embryos from the experiments ranged from ~250 to ~2200 units.


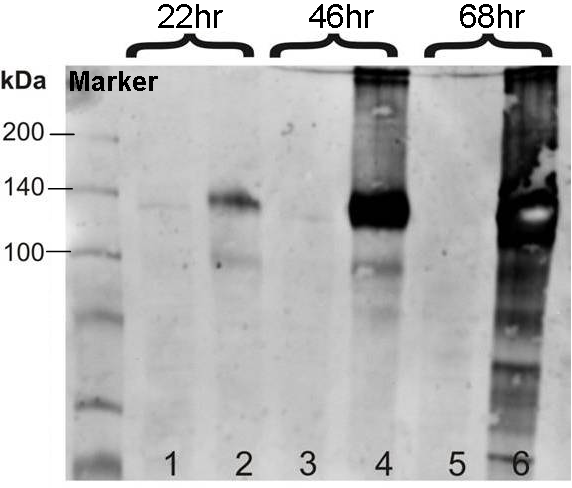


**Figure S5.** Western blots of protein extracts from Sf9 cells with recombinant Abcb4 from zebrafish. Recombinant proteins were obtained with the baculovirus expression system. For protein detection we used the C219 antibody that was raised against a conserved epitope in mammalian Abcb1. Total protein of baculovirus-infected Sf9 cells was isolated at three different time points, subjected to SDS-PAGE and blotted to nitrocellulose membranes. Concentrations of the primary and secondary antibodies were 1:500 and 1:1000, respectively. Lanes 1, 3 and 5 on each blot show negative controls (protein from non-infected Sf9 cells isolated in parallel to protein from infected cells), lanes 2, 4 and 6 show the blots of isolates from Abcb4 baculovirus-infected cells. The size of the intact Abcb4 protein is ca.140 kDa. Each lane was loaded with 2 µg protein.


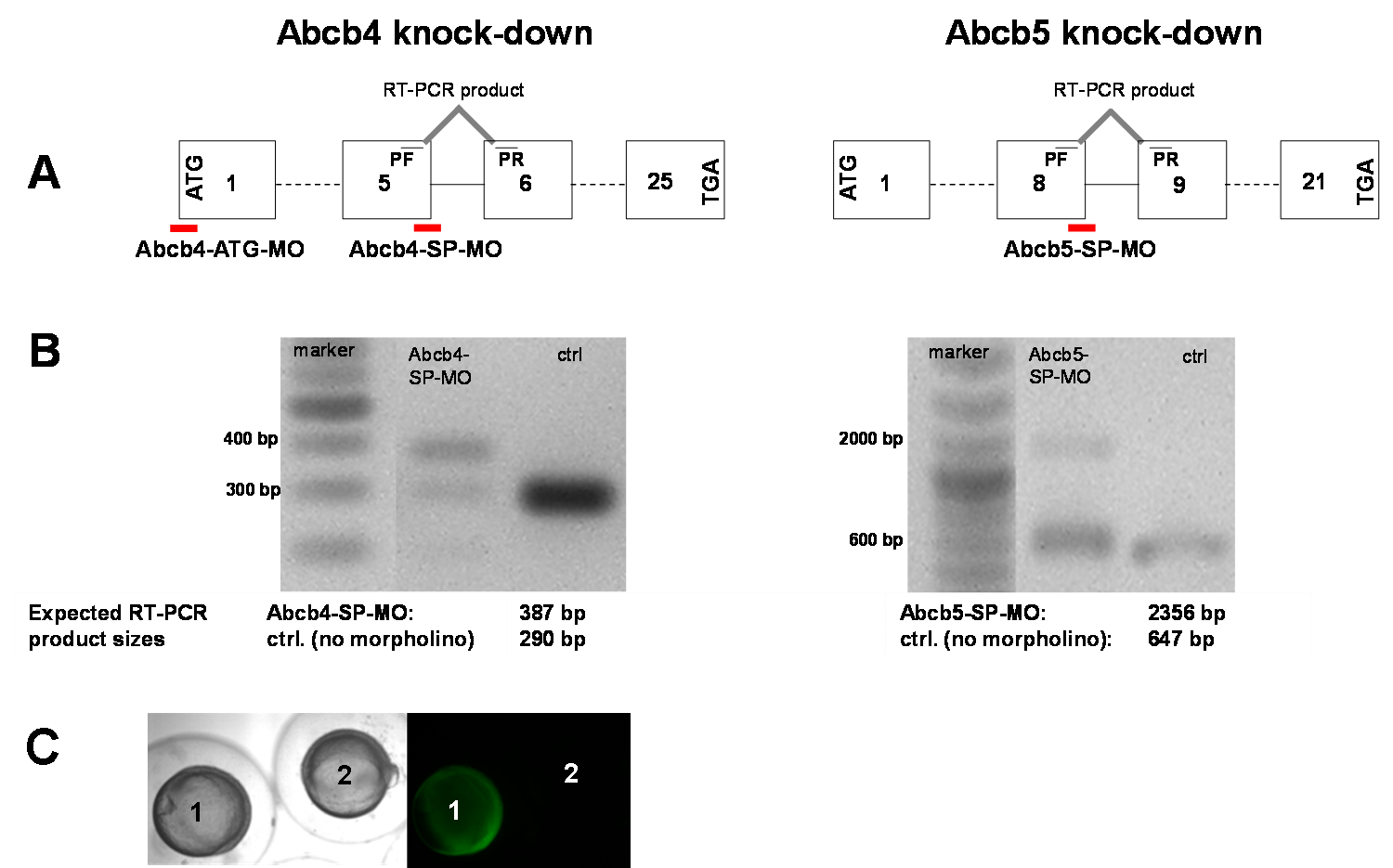


**Figure S6.** Proof of functionality of the used morpholinos.

**A**) Schemas of *abcb4* and *abcb* genes with positions of exon-intron binding sites of splice-blocking morpholinos (Abcb4-SP-MO, Abcb5-SP-MO, 0.5 mM each) and of the translation-blocking morpholino binding site (Abcb4-ATG-MO, 0.0625 mM). Also indicated are positions of RT-PCR primer binding (PF: forward, PR: reverse). RT-PCR was used to confirm functionality of splice-blocking morpholinos.

**B**) Images of ethidium bromide-stained agarose gels with RT-PCR products of *abcb4* and *abcb5* segments obtained from total RNA of knock-down and control (ctrl) zebrafish embryos (24 hpf). Indicated below the images are expected sizes of RT-PCR products. Sizes of the obtained RT-PCR products were according to expected sizes and confirm miss-splicing of *abcb4* and *abcb5* pre-mRNAs by morpholinos Abcb4-SP-MO and Abcb5-SP-MO.

**C**) Micrographs of zebrafish embryos (7 hpf) upon injection of a GFP mRNA containing the Abcb4-ATG-MO binding site in front of the GFP coding sequence. Embryo 1 was injected with this GFP-RNA construct only and GFP fluorescence was detectable. Embryo 2 was co-injected with the GFP-RNA construct and Abcb4-ATG-MO translation-blocking morpholino. As can be seen from the fluorescence micrograph, GFP fluorescence in embryo 2 was reduced compared to embryo 1, confirming functionality of Abcb4-ATG-MO.
